# Distinct Basal Gut Microbiota Profiles Are Associated with Strain-Specific Lung Responses to *Aspergillus fumigatus*

**DOI:** 10.3390/biology15141132

**Published:** 2026-07-11

**Authors:** Dusanka Popovic, Ivana Mirkov, Dina Tucovic, Aleksandra Popov Aleksandrov, Anastasija Malesevic, Stanislava Stanojevic, Jasmina Glamoclija, Maja Tolinacki, Milica Zivkovic, Jelena Kulas

**Affiliations:** 1 Immunotoxicology Group, Department of Ecology, Institute for Biological Research “Sinisa Stankovic”—National Institute of the Republic of Serbia, University of Belgrade, 11000 Belgrade, Serbia; dusanka.popovic@ibiss.bg.ac.rs (D.P.); mirkovi@ibiss.bg.ac.rs (I.M.); dina.mileusnic@ibiss.bg.ac.rs (D.T.); aleksandrap@ibiss.bg.ac.rs (A.P.A.); anastasija.malesevic@ibiss.bg.ac.rs (A.M.); stanislava.stanojevic@ibiss.bg.ac.rs (S.S.); 2 Department of Plant Physiology, Institute for Biological Research “Sinisa Stankovic”—National Institute of the Republic of Serbia, University of Belgrade, 11000 Belgrade, Serbia; jasna@ibiss.bg.ac.rs; 3 Group for Probiotics and Microbiota-Host Interaction, Department of Microbiology and Plant Biology, Institute of Molecular Genetics and Genetic Engineering, University of Belgrade, 11000 Belgrade, Serbia; maja.tolinacki@imgge.bg.ac.rs (M.T.); milica.zivkovic@imgge.bg.ac.rs (M.Z.)

**Keywords:** Albino Oxford (AO) rat strain, Dark Agouti (DA) rat strain, antibiotics, *Aspergillus fumigatus* lung infection, gut microbiota, lung microbiota, next generation sequencing, inflammation

## Abstract

Background: Antibiotic-associated gut dysbiosis affects lung health and contributes to disease progression, but the individual response to antibiotics, which leads to host-specific reactions to the same respiratory insult, is not fully understood. Methods: Before infection with *Aspergillus fumigatus*, Albino Oxford (AO) and Dark Agouti (DA) rats received a mixture of antibiotics with poor systemic absorption to induce microbial perturbation only in the gut, and lung and gut tissue and colonic contents were analyzed. Results: Treatment with antibiotics induced gut inflammation in both rat strains, which was more pronounced in AO rats, and a disturbance in lung immunity was observed only in DA rats. Antibiotic-associated gut microbiota disturbance revealed rat strain-specific bacterial profiles (increased Gammaproteobacteria in DA and decreased Coriobacteriia in AO rats). Although gut dysbiosis increased the risk of lung *A. fumigatus* infection in both rat strains, a higher fungal burden was detected in the lungs of AO rats, whereas lymph node cell activation and lung tissue inflammation were observed in DA rats. Conclusions: Rat strain differences in response to antibiotic- and/or infection-induced gut dysbiosis suggest that basal gut microbiota may influence the lung immune response during *A. fumigatus* disease, highlighting the importance of the interplay of genotype-specific host gut microbial community with the environmental constraints in shaping individual immune makeup.

## Simple Summary

Antibiotic-associated changes in the gut microbial community (dysbiosis) lead not only to perturbations in gut immunity, but also to immune changes in distant lung tissue. It is still not known whether an individual response to antibiotics may result in a host-specific reaction to the same respiratory pathogen. To address this question, Albino Oxford and Dark Agouti rat strains were orally treated for thirty days with a mixture of antibiotics with poor systemic absorption to induce microbial perturbation only in the gut and then infected with the fungus *Aspergillus fumigatus*. Treatment with antibiotics alone induced gut dysbiosis and inflammation in both rat strains, whereas lung inflammation was observed only in Dark Agouti rats. Subsequent *A. fumigatus* infection produced a higher fungal burden in the lungs of Albino Oxford rats, whereas lymph node cell activation and lung tissue inflammation were still observed only in Dark Agouti rats. These findings suggest that basal gut microbiota influences lung immune responses during *A. fumigatus* infection and may determine individual differences in both the response to antibiotic treatment and the response to infection.

## 1. Introduction

Balanced gut microbiota is a key factor in maintaining host health. This influence is exerted through processes such as immune system maturation, nutrient metabolism, vitamin synthesis, gut barrier maintenance, pathogen exclusion, etc. [1]. However, the effects of a disrupted gut community are not only local, but also manifest in distant organs such as the skin, brain, and lungs. This gut–distal organ communication is established through bi- or multidirectional axes via neural, endocrine, humoral, and metabolic pathways [2]. The crosstalk between the gut and lungs is known as the gut–lung axis. Gut microbiota metabolites such as short-chain fatty acids, bile acids, and amino acids play a protective role in lung health, regulating pulmonary inflammation and supporting proper immune responses to various lung insults [3]. In that context, a balanced gut community is crucial for the host’s capacity to resist infections [4]. Numerous studies have shown that disturbances in gut microbiota are associated with respiratory diseases such as asthma [5,6,7], chronic obstructive pulmonary disease [8], cystic fibrosis [9], and lung viral [10,11] or bacterial infections [4,12]. A dysbiotic gut characterized by decreased microbial diversity, increased relative abundance of pro-inflammatory *Prevotella* and *Enterococcus,* and reduced levels of beneficial Ruminococcaceae and Bifidobacteriaceae has been noted in pediatric populations with *Mycobacterium tuberculosis* infection [13]. In adult patients with pulmonary tuberculosis (PTB), altered gut microbiota (with enrichment of *Bacteroides* and *Prevotella* and depletion of *Blautia* and *Bifidobacterium*) has also been observed [14]. Significant differences in gut community composition and metabolic pathways between PTB patients and healthy controls were reported in the studies of Ye and coworkers [15] and Wang and coworkers [16]. In addition, gastrointestinal symptoms such as abdominal pain, nausea, vomiting, and diarrhea have been reported in lung viral infections as well [10]. In the murine gut, both respiratory syncytial and influenza viruses caused an increase in the ratio of Bacteroidetes to Firmicutes phyla [17]. A reduction in alpha diversity and an increase in the relative abundance of *Escherichia coli* and *Enterococcus faecium* were characteristic of patients with influenza virus infection [11]. Furthermore, a higher relative abundance of opportunistic pathogens and lower abundance of beneficial bacteria were observed in COVID-19 patients compared with healthy controls [18,19]. Data on the gut response to lung fungal infection also exist. Pulmonary *Cryptococcus neoformans* infection in mice, a model of cryptococcosis, induced gut dysbiosis characterized by decreased abundance of beneficial commensals and increased abundance of opportunistic pathogens [20]. Similarly, disruption of gut homeostasis caused by the opportunistic fungus *Aspergillus fumigatus* was confirmed by our group [21,22].

Since the gut–lung axis is based on a complex bidirectional relationship, it is often difficult to determine whether disturbances in the gut community are the cause or the consequence of lung pathology [23]. The most common approach for studying the effects of gut microbiota on the development of lung diseases is the use of antibiotics that disrupt the gut. Experimental studies investigating the influence of gut microbiota in lung bacterial [24,25,26,27] or viral [28,29] infections often rely on antibiotics with high systemic diffusion. Because antibiotic penetration into tissue disturbs both gut and lung microbiota [26,27], it is difficult to evaluate to what extent gut microbiota itself contributes to the pathogenesis of lung diseases.

Apart from several studies that examined the effect of lung fungal infections on intestinal microbiota (lung–gut axis) [20,21,22], little is known about how gut microbiota affects the lung immune response during infection (gut–lung axis). In the present study, using an experimental model of lung infection caused by the fungus *A. fumigatus* in Dark Agouti (DA) and Albino Oxford (AO) rat strains, we investigated the effects of disrupted gut microbiota during lung aspergillosis. To examine the role of gut bacterial microbiota in the lung immune response to *A. fumigatus*, we used oral antibiotics with poor systemic absorption. Since our previous study showed that DA and AO rats exhibit quantitatively different immune reactions to the same respiratory insult [30] and that alteration of gut microbiota in response to pulmonary infection depends on the basal gut community [31], we aimed to determine whether rat strain differences in gut bacterial composition lead to different gut microbiota responses to antibiotics, which may, in turn, cause a strain-specific lung response to the same fungus.

## 2. Materials and Methods

### 2.1. Experimental Animals

Ten- to twelve-week-old male Dark Agouti (DA) and Albino Oxford (AO) rats were obtained from the breeding colony of the Institute for Biological Research “Sinisa Stankovic” (IBISS), Belgrade, Serbia. The animals were housed in a controlled environment (22 ± 2 °C, relative humidity 60%, and under a 12:12 h light:dark cycle) in standard cages, with *ad libitum* access to conventional rodent food (Veterinarski zavod Subotica, Serbia containing 3.2% fat, 49.3% carbohydrates, 20.3% proteins, and 12.9% fibers, energetic value 1405 KJ/100 g) and tap water until the beginning of the experiment.

### 2.2. Fungal Culture Conditions and Conidia Preparation

As previously described [22,31], a human isolate of *A. fumigatus* (obtained from the Institute of Public Health of Serbia “Dr. Milan Jovanovic-Batut”) was grown on Sabouraud maltose agar (SMA, Torlak, Belgrade, Serbia) for seven days to produce conidia. Conidia were collected by flooding the surface of agar slants with pyrogen-free, sterile physiological saline (Hemofarm, Vrsac, Serbia), counted using an improved Neubauer hemocytometer, and adjusted to a concentration of 10^7^/mL.

### 2.3. Experimental Procedure

For both DA and AO rat strains, half of the experimental animals received distilled water for 30 days, while the other half received distilled water containing antibiotics with poor systemic absorption for 30 days. The antibiotic mixture consisted of a combination of broad-spectrum aminoglycoside antibiotics, neomycin sulfate and gentamicin sulfate, acting against Gram-negative bacteria, each prepared at a concentration of 0.5 g/L, and the glycopeptide antibiotic vancomycin, acting against Gram-positive bacteria, prepared at a concentration of 0.25 g/L. Neomycin and gentamicin were purchased from Veterinarski zavod Subotica (Subotica, Serbia), while vancomycin was purchased from ALVOGEN Pharma (Plandiste, Serbia). Fresh water and antibiotic solutions were prepared twice a week, and the consumption of each was measured. Body mass was measured once a week.

On day 31, animals from the water-treated and antibiotic-treated groups were anesthetized (Zoletil 100, Virbac, Carros, France) and intratracheally administered either with 50 μL of saline or with 50 μL of saline containing 10^7^ conidia/mL of *A. fumigatus*.

A total of 46 animals were assigned to four experimental groups for each rat strain: water-treated uninfected group (*n* = 5), antibiotic-treated uninfected group (*n* = 6), water-treated infected group (*n* = 6), and antibiotic-treated infected group (*n* = 6) for DA and AO rat strains. For microbiota analysis, 35 animals were assigned to the following groups: water-treated uninfected group (*n* = 4), antibiotic-treated uninfected group (*n* = 4), water-treated infected group (*n* = 5), and antibiotic-treated infected group (*n* = 5) for DA, and water-treated uninfected group (*n* = 4), antibiotic-treated uninfected group (*n* = 4), water-treated infected group (*n* = 5), and antibiotic-treated infected group (*n* = 4) for AO rats.

Animals were euthanized on day 3 post-infection (p.i.) by intraperitoneal injection of 15 mg/kg b.w. of Zoletil 100 (Virbac, Carros, France).

### 2.4. Tissue Sample Collection and Preparation

Blood was withdrawn from the abdominal aorta with a syringe containing 5 U/mL heparin, and hematological parameters were determined automatically using a Siemens ADVIA 120 flow cytometer (Tarrytown, NY, USA) with commercially available reagents. Following centrifugation, plasma was collected for cytokine level determination.

The lungs with regional mediastinal lymph nodes and intestinal segments with mesenteric lymph nodes were aseptically removed. The lungs were divided into the right lung (for preparation of tissue homogenates) and the left lung (for metagenomic DNA extraction) and stored at −80 °C until use. To avoid cross-contamination of lung samples for microbiota analysis, aseptic procedures, processing and storage were prioritized to minimize contaminant presence. However, as no negative control was included, we acknowledge this as a limitation of the study. Mediastinal and mesenteric lymph nodes were mechanically dissociated by passage through a nylon mesh (70 μm nylon, BD Bioscience, Bedford, MA, USA), washed, and the cell number was determined with an improved Neubauer hemocytometer (the number of viable cells exceeded 90% according to trypan blue exclusion). The cells were used for analysis of cytokine gene expression. The duodenum was excised as a 5 cm segment immediately below the pyloroduodenal junction, the ileum was removed as a 3 cm segment just above the ileocecal junction, and the cecum and colon were isolated at full length and colonic contents (feces) were extruded. Intestinal segments were washed through the lumen with ice-cold, nonpyrogenic physiological saline, weighed, snap-frozen in liquid nitrogen, and stored at −80 °C until use.

Right lung tissue and intestinal tissue samples were homogenized on ice with an IKA T18 Basic Homogenizer (IKA Works Inc., Wilmington, NC, USA) in ten volumes of sucrose buffer consisting of 10 mM Tris-HCl (Tris (hydroxymethyl) aminomethane, Serva Feinbiochemica, Heidelberg, Germany), pH 7.6, 1 mM N, N, N′, N′-ethylenediaminetetraacetic acid (EDTA) disodium salt dihydrate (USB Corporation, Cleveland, OH, USA), 250 mM sucrose, and 1 mM peptidase inhibitor phenylmethanesulfonyl fluoride (PMSF, Sigma Chemical Co., St. Louis, MO, USA). Homogenates were sonicated (3 × 15 s on ice at 30% of maximum intensity amplitude) with a laboratory sonicator (Bandelin electronic, UW 2070, Berlin, Germany) and then centrifuged (10,000× *g* for 20 min at 4 °C) to obtain supernatants that were used to determine cytokine levels and oxidative stress parameters. The left lung tissue and colonic content were used for bacterial DNA extraction.

To determine the infectious burden in *A. fumigatus*–infected lungs, right lung tissue was weighed and homogenized with an IKA T18 Basic Homogenizer (IKA Works Inc., Wilmington, NC, USA) in 5 mL of physiological saline, seeded on SMA with streptomycin sulfate, and incubated for 48 h at 37 °C. The number of *A. fumigatus* colonies in infected lungs was counted, and the number of colony-forming units (CFU/g) was calculated.

### 2.5. Tissue DNA Extraction and Sequencing

Metagenomic DNA was extracted from frozen lung and fecal samples using the ZR Tissue DNA Miniprep and Fecal/Soil Microbe Miniprep Kit (Zymo Research Corp., Irvine, CA, USA), according to the manufacturer’s instructions. DNA concentration was measured with a Qubit™ fluorometer (Thermo Fisher/Invitrogen, Waltham, MA, USA). All samples were diluted to 12 ng/μL in a final volume of 20 μL and sent to Novogene Company (Cambridge, UK) for library preparation and 16S rRNA amplicon sequencing of the V3–V4 hypervariable region. Paired-end sequencing was performed on an Illumina NovaSeq platform. After barcode, adapter, and primer removal, paired-end reads were analyzed and merged using FLASH (V1.2.7) [32]. High-quality reads and sufficient sequencing depth were confirmed by flat rarefaction curves and a high coverage index for each sample. Quality filtering of the raw tags was performed under specific filtering conditions [33] according to QIIME (V1.7.0) [34]. For species annotation at each taxonomic rank, QIIME Version 1.7.0 [35] in the Mothur method was used against the SSU rRNA database of the SILVA 138 Database [36]. Alpha and beta diversity analyses were performed based on normalized operational taxonomic unit (OTU) data. Alpha diversity, as a measure of microbiota diversity, was expressed through three indices: Observed species, Shannon, and Chao1. Beta diversity, as a measure of similarity or dissimilarity between communities, was visualized using Principal Coordinates Analysis (PCoA) using Jensen–Shannon distance and was expressed through ANOSIM and Adonis, performed by Novogene Technology Co., Ltd. (Cambridge, UK). Linear discriminant analysis effect size (LEfSe) was performed using the Galaxy server [37] (LEfSe platform from Galaxy). Although off-target amplification may occur during 16S rRNA gene sequencing, particularly in low–biomass samples such as lung tissue, host-reads statistics were not quantified in our standard V3–V4 amplicon-sequencing workflow. To minimize the inclusion of non-target sequences, quality control processing was performed prior to downstream analysis.

Data on Reads obtained per sample (Table S1), Quality control data for FASTQ files (Table S2), and The primer sequences table (Table S3) are provided in Supplementary File S1.

### 2.6. Determination of Parameters of Oxidative Stress and Antioxidative Defense

Lung and gut tissue homogenates were used to determine reduced glutathione (GSH) and malondialdehyde (MDA) content, as well as glutathione S-transferase (GST, EC 2.5.1.18) and catalase (CAT, EC 1.11.1.6) activity.

GSH content was measured in the deproteinized supernatant of samples prepared in 10% sulphosalicylic acid [38], using 5,5-dithio-bis-(2-nitrobenzoic acid) (DTNB) in Tris–Cl (pH 8.9) and reduced glutathione as the standard. Absorbance was monitored spectrophotometrically at 412 nm, and the data are expressed as μM/mg of precipitated protein.

GST activity was determined by measuring the rate of formation of the DNCB-GSH complex catalyzed by this enzyme [39]. The reaction mixture consisted of the sample (10 μL), deionized water, 1-chloro-2,4-dinitrobenzene (DNCB, Sigma Chemical Co., St. Louis, MO, USA) dissolved in 95% ethanol, phosphate buffer (pH 6.5), and glutathione (GSH, Fluka Chemie, Buchs, Switzerland). The increase in absorbance was monitored spectrophotometrically at 340 nm every 30 s for 180 s at 25 °C. The GST activity unit was expressed as the number of nanomoles of the DNCB–GSH complex formed per minute (U = nM DNCB–GSH/min) per milligram of protein (U/mg protein).

Catalase (CAT, EC 1.11.1.6) activity was measured following the method of Beutler [40]. Samples (10 μL) were mixed with 50 μL Tris–EDTA buffer (pH 8.8) and 1 mL of hydrogen peroxide (H_2_O_2_, Zorka Farma, Sabac, Serbia), and the change in absorbance was recorded for 3 min (every 30 s) at 25 °C. One unit of CAT activity was defined as the amount of enzyme required to decompose 1 mM H_2_O_2_ per minute. The rate of H_2_O_2_ decomposition was measured spectrophotometrically at 240 nm using a Shimadzu UV-1800. Enzyme activity was expressed as units per milligram of protein (U/mg protein).

For the evaluation of lipid peroxidation and formation of malondialdehyde (MDA), the method of Villacara et al. [41] was used. Samples were mixed with 2-thiobarbituric acid (TBA), and trichloroacetic acid (TCA) reagent (both from Sigma Chemical Co., St. Louis, MO, USA), and Tris–HCl (pH 7.4), and heated for 60 min at 95 °C. The absorbance of the obtained supernatant was measured at 535 nm using a spectrophotometer (Shimadzu Corporation, Lakewood, CA, USA). MDA content in the homogenates was determined using a standard curve generated from known amounts of MDA (Sigma Chemical Co., St. Louis, MO, USA). Data were expressed as nM MDA/mg protein.

Protein concentration was determined by the Lowry assay [42], in which 20 μL tissue homogenate was mixed with 300 μL reagent C [2% sodium carbonate (Na_2_CO_3_, Carlo Erba, Milano, Italy) in 0.1 M sodium hydroxide (NaOH, LachNer, Neratovice, Czech Republic), 1% copper sulfate pentahydrate (CuSO_4_ × 5H_2_O, Zorka, Sabac, Serbia), and 2% potassium sodium tartrate (KNaC_4_H_4_O_6_ × 4H_2_O, Alkaloid, Skopje, North Macedonia)] and 60 μL of 1 × Folin–Ciocalteu phenol reagent (Merck, Darmstadt, Germany). Absorbance was measured at 670 nm using a Shimadzu UV-1800 spectrophotometer (Kyoto, Japan), and protein concentration was calculated using bovine serum albumin (BSA, AppliChem, Darmstadt, Germany) as a reference.

### 2.7. Determination of Myeloperoxidase (MPO) Activity

Myeloperoxidase activity was evaluated in lung homogenates based on the oxidation of o-dianisidine dihydrochloride [43]. MPO was measured by adding 33 μL of supernatant to 967 μL of substrate solution [0.167 mg/mL *o*-dianisidine dihydrochloride (Sigma Chemical Co., St. Louis, MO, USA) and 0.0005% H_2_O_2_ in 50 mM potassium phosphate buffer, pH 6.0]. Absorbance was measured at 450 nm with an ELISA 96-well plate reader (GRD, Rome, Italy). MPO activity was calculated with reference to a standard curve generated with known amounts of MPO (Sigma Chemical Co., St. Louis, MO, USA). Values are presented as MPO units (U)/mL.

### 2.8. Cytokine Determination by ELISA

Supernatants of lung and intestinal tissue samples were used to determine cytokine production with commercially available ELISA kits: interleukin (IL)-1β, IL-6, tumor necrosis factor (TNF), and IL-10 (R&D Systems, Minneapolis, MN, USA); IL-17 and interferon (IFN)-γ (eBioscience Inc., San Diego, CA, USA). Cytokine concentrations were calculated from a standard curve constructed with known amounts of recombinant cytokines provided by the manufacturer. Results are expressed as pg/mL.

### 2.9. Cytokine Determination by Reverse Transcription Real-Time Polymerase Chain Reaction (RT-PCR)

RNA (1 μg) was isolated from mediastinal and mesenteric lymph node cells using a mi-Total RNA Isolation Kit (Metabion, Martinsried, Germany) and reverse transcribed using random hexamer primers and MMLV (Moloney Murine Leukemia Virus) reverse transcriptase (Fermentas, Vilnius, Lithuania), following the manufacturer’s instructions. According to the recommendations, the prepared cDNAs were amplified using Power SYBR^®^ Green PCR Master Mix (Applied Biosystems, Foster City, CA, USA) in a total volume of 20 μL on a QuantStudioTM 3 Real-Time PCR Instrument (96-well, 0.2 mL) (Applied Biosystems). Thermocycler conditions were as follows: an initial step at 50 °C for 5 min, followed by a step at 95 °C for 10 min, and a subsequent two-step PCR program at 95 °C for 15 s and 60 °C for 60 s for 40 cycles. The PCR primers used in the study are listed in Table 1.

Quantitative differences in gene expression levels were assessed using QuantStudioTM Design & Analysis Software v1.4.3 (Applied Biosystems), and the results were calculated as 2^−ΔCt^, where −ΔCt represents the difference between the threshold cycle (Ct) values of a specific gene and the endogenous control (β-actin). Results are expressed as mRNA expression (2^−ΔCt^).

### 2.10. Statistical Analysis

All statistical analyses of the results, except for microbiota, were performed using STATISTICA 7.0 (StatSoft Inc., Tulsa, OK, USA). Normality of the data was assessed using the Shapiro–Wilk test. Most of the variables were normally distributed, whereas variables that did not meet the normality assumption were transformed. For multiple group comparisons, one-way analysis of variance (ANOVA) was performed on the original and transformed data, followed by Tukey’s post hoc test. For differential abundance analysis of class-level fecal microbiota taxa (only taxa with relative abundance greater than 0.01% in at least one sample were included) in uninfected water-treated DA and AO rats, we used the nonparametric Kruskal–Wallis test followed by Dunn’s post hoc test. *p* values were adjusted with the Benjamini–Hochberg false discovery rate (FDR) procedure, and effect size was estimated using η^2^ (H). To identify clusters in the gut community between uninfected water-treated DA and AO rats, we applied PCoA based on Jensen–Shannon distances with PAM clustering. For comparisons between two groups, a nonparametric Wilcoxon rank-sum test was used. To identify the top 10 drivers of community variation, we calculated Spearman correlations between genus-level relative abundances and the first principal coordinate (PC1), with the corresponding *p* values adjusted using the Benjamini–Hochberg FDR procedure. To represent differences in gut and lung microbiota community structure between groups, we performed PCoA based on Jensen–Shannon distances followed by Adonis and ANOSIM statistical tests. LEfSe analysis was used to identify differentially abundant taxa at the genus level, using the Kruskal–Wallis test followed by pairwise Wilcoxon tests and linear discriminant analysis (LDA threshold of 2.0) to estimate effect size. The level of statistical significance was set at *p* < 0.05. All microbiota analyses were performed in R (version 4.5.2), with the exception of LEfSe, which was performed using the Huttenhower Lab Galaxy server [37]. Complete statistical analyses are provided in Supplementary File S2: Daily fluid and antibiotic intake in uninfected DA and AO rats (Table S1); Alpha diversity in the lungs and feces of uninfected DA and AO rats (Table S2); Alpha diversity in the lungs and feces of infected DA and AO rats (Table S3); The effect of antibiotic treatment on the relative abundance of fecal bacteria at the class level in uninfected DA and AO rats (Table S4); Top 10 drivers of community variations in uninfected DA and AO rats (Table S5); Immune homeostasis and oxidative parameters in the lungs, gut, and plasma of uninfected DA and AO rats (Table S6); The effect of antibiotic treatment on the fungal burden in the lungs of infected DA and AO rats (Table S7); and Immune homeostasis and oxidative parameters in the lungs, gut, and plasma of infected DA and AO rats (Table S8).

## 3. Results

### 3.1. Antibiotic Administration Caused Bacterial Dysbiosis in the Gut but Not in the Lungs

To determine the extent to which treatment with oral antibiotics affects lung and gut microbiota and thereafter the immune response, a mixture of oral antibiotics with poor systemic absorption (neomycin 0.5 g/L, gentamicin 0.5 g/L, and vancomycin 0.25 g/L) was given to DA and AO rats for 30 days.

During this period, no differences in body mass were observed between uninfected animals that drank water and those given water with antibiotics, but total fluid consumption was increased in animals that received antibiotics (Table 2). Daily antibiotic intake (calculated based on concentration of antibiotics, rat body mass (BM) and daily fluid consumption) was not different between uninfected DA and AO rat strains (Table 2).

**Figure 1:**
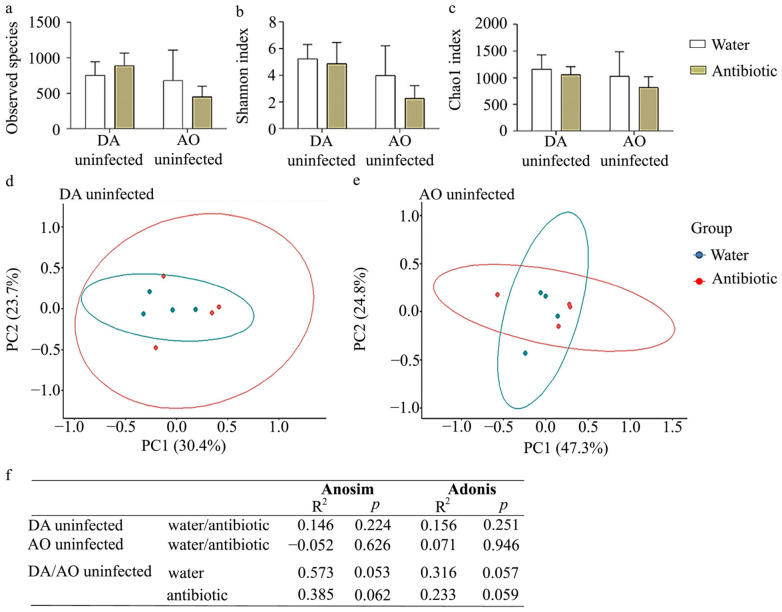
Effect of 30-day oral consumption of antibiotic solution on lung bacterial microbiota in uninfected DA and AO rats: alpha diversity expressed through Observed species (**a**), Shannon index (**b**), and Chao1 index (**c**), and beta diversity expressed through Principal Coordinates Analysis (PCoA), based on Jensen–Shannon divergence at the genus level, in uninfected DA (**d**) and AO (**e**) rats. Differences in bacterial community in the lungs of uninfected DA and AO rats treated with antibiotics expressed through Adonis and ANOSIM are depicted in (**f**). Results in (**a**–**c**) are expressed as mean ± SD.

As expected, alpha diversity analysis revealed no statistically significant differences in lung microbiota richness (Observed species and Chao1 index) or evenness (Shannon index) following treatment with antibiotics with poor systemic absorption in uninfected DA and AO rats (Figure 1a–c). To visualize the effect of antibiotic administration on lung microbiota, we performed Principal Coordinates Analysis (PCoA) at the genus level, which showed no clear separation between rats that drank water and those treated with antibiotic solution in either strain (Figure 1d,e). This was also confirmed by the ANOSIM and Adonis statistical tests (Figure 1f).

Although higher R values generally indicate greater community variation and group separation, respectively, this did not reach statistical significance in our data, which was also supported by the PCoA visual representation. The absence of significance may reflect the small sample size and the greater heterogeneity characteristic of the lung microbiota community.

**Figure 2:**
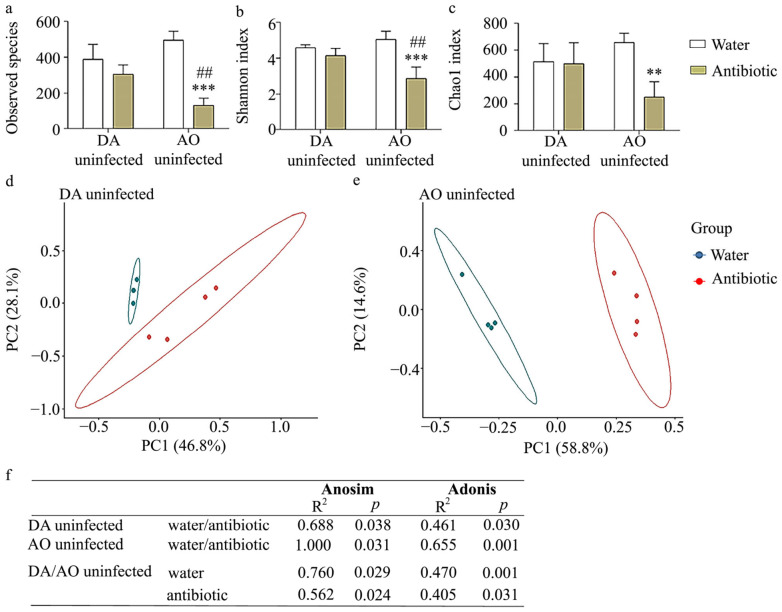
Effect of 30-day oral consumption of antibiotic solution on fecal bacterial microbiota in uninfected DA and AO rats: alpha diversity expressed through Observed species (**a**), Shannon index (**b**), and Chao1 index (**c**); and beta diversity expressed through Principal Coordinates Analysis (PCoA), based on Jensen–Shannon divergence at the genus level, in uninfected DA (**d**) and AO (**e**) rats. Differences in fecal bacterial community in uninfected DA and AO rats treated with antibiotics expressed through Adonis and ANOSIM are shown in (**f**). Results in (**a**–**c**) are expressed as mean ± SD. Statistically significant differences: ** *p* < 0.01 and *** *p* < 0.001 vs. water; ^##^ *p* < 0.01 vs. DA.

On the other hand, the effects of antibiotic administration were more pronounced in the gut. While no changes in alpha diversity were observed in DA rats, a significant decrease in all three indices (Observed species, Shannon index, and Chao1 index) was noted in the gut of AO rats as a consequence of antibiotic treatment (Figure 2a–c). However, antibiotic administration induced changes in the gut microbiota community structure of both uninfected DA and AO rat strains, according to PCoA (Figure 2d,e), which was confirmed by the Adonis and ANOSIM statistical analyses (Figure 2f).

To investigate more detailed taxonomic shifts, we analyzed changes in the relative abundance of bacteria at the class level (Supplementary File S2, Table S4). Although the abundance of Coriobacteriia and Gammaproteobacteria was similar between uninfected DA and AO rats that drank water for 30 days, antibiotic administration caused strain-specific responses at these taxonomic levels. In uninfected DA rats, antibiotics stimulated the growth of Gammaproteobacteria, while reducing Coriobacteriia in uninfected AO rats.

**Figure 3:**
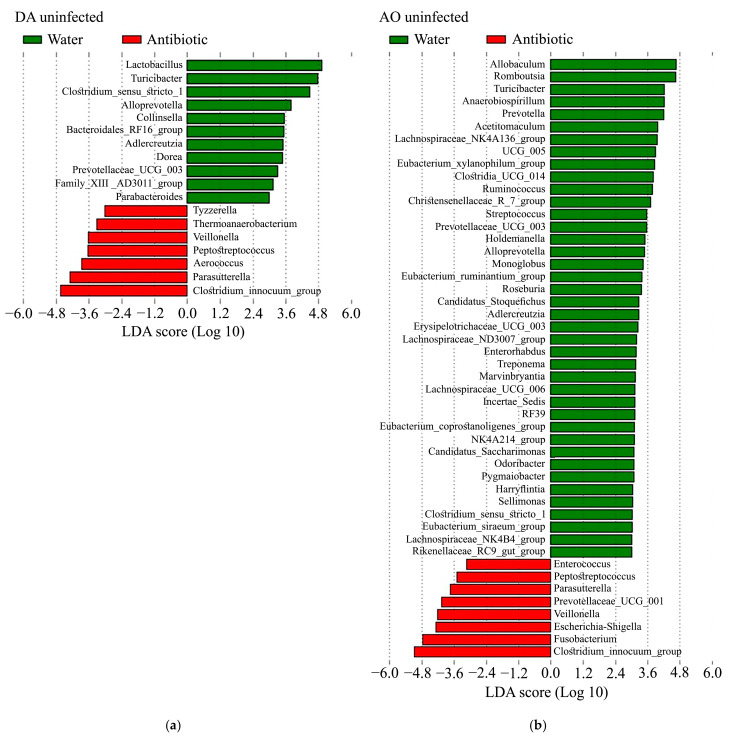
Effect of 30-day oral consumption of an antibiotic solution on gut microbial biomarkers, presented using the Linear Discriminant Analysis Effect Size (LEfSe) method at the genus level in uninfected DA (**a**) and AO (**b**) rats.

To identify biomarkers related to each group, we performed Linear Discriminant Analysis Effect Size (LEfSe) (Figure 3). The DA water-treated uninfected group was dominated by genera mainly from the classes Bacteroidia and Clostridia (36.4% and 27.2%, respectively), with members of the normal gut microbiota positively associated with gut health, whereas the antibiotic-treated group showed an overabundance of bacteria belonging to the class Clostridia (57.1%), often described in dysbiotic gut (Figure 3a). Furthermore, LEfSe analysis revealed a higher number of discriminative genera in the AO water-treated compared with the antibiotic-treated group, suggesting lower microbial diversity after antibiotic treatment. While more than half (60%) of differentially abundant genera in the water-treated group were members of the class Clostridia, mainly recognized as gut commensals, antibiotic administration caused an overabundance of bacteria mostly from the classes Clostridia and Bacilli (25% from each), many of which have pathogenic potential (Figure 3b). However, it appears that some of the discriminative genera were common to both rat strains in the antibiotic-treated groups. Higher relative abundance of *Peptostreptococcus*, *Parasutterella*, *Veillonella*, and the *Clostridium innocuum group* suggests that these genera were a shared microbial signature of antibiotic-induced dysbiosis, despite existing differences in the microbiota response to the same treatment.

**Figure 4:**
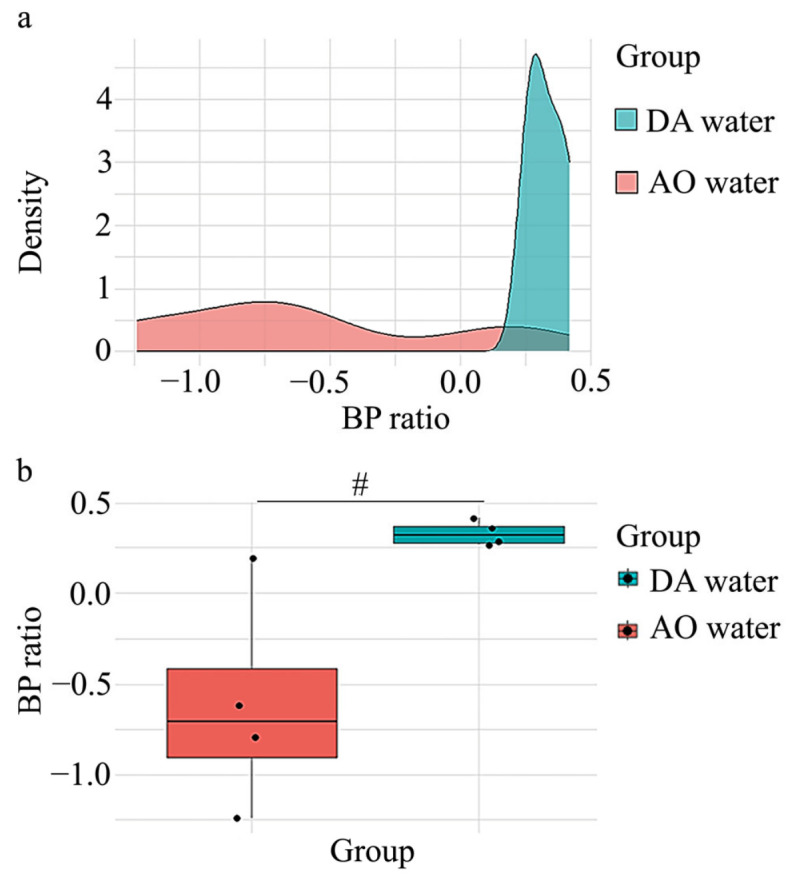
Distribution of the continuous *Bacteroides-Prevotella* (*B/P*) gradient (**a**) and *B/P* ratio (**b**) in water-treated uninfected DA (blue) and AO (red) rats. Statistically significant differences: # *p* < 0.05.

Because the initial state of the microbiota determines the effect of antibiotics [44,45] and may even depend on enterotype [46], we examined whether our water-treated uninfected DA and AO animals followed a continuous gradient that might explain the observed differences in response to antibiotics. Our groups do not follow a strict enterotype, but their microbiota is compatible with a gradient distribution (Figure 4a). Microbiota from water-treated uninfected DA rats exhibited a more consistent, denser microbial population, with a higher *Bacteroides*-to-*Prevotella* (*B/P*) ratio shifted toward *Bacteroides*, whereas microbiota from water-treated uninfected AO rats showed a more dispersed composition, reflecting greater heterogeneity, with a lower *B/P* ratio shifted toward *Prevotella* (*p* = 0.029, Figure 4b).

**Figure 5:**
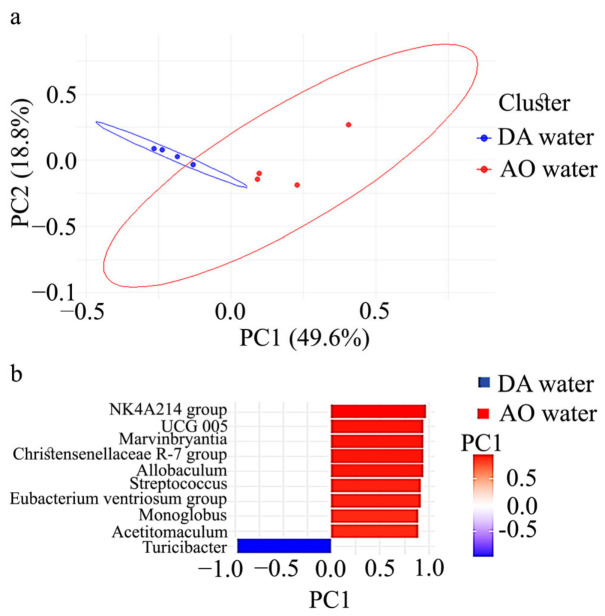
Beta diversity of fecal bacterial microbiota expressed through Principal Coordinates Analysis (PCoA) based on Jensen–Shannon distances with PAM clustering at the genus level (**a**), and bar plot with the top 10 PCoA drivers of community variation (**b**), in water-treated uninfected DA (blue) and AO (red) rats.

The PCoA plot with PAM clustering and statistical tests also showed clear separation between the microbial communities of water-treated uninfected DA and AO rat strains (Figure 5). Compositional variation at the class level between water-treated uninfected DA and AO rats was reflected in a higher abundance of Actinobacteria in DA animals compared with AO (0.034 ± 0.006 in DA vs. 0.001 ± 0.000 in AO, *p* = 0.0178). To determine the main drivers of community variation that provide better resolution of microbial differences, we identified the genera responsible for the observed differences between water-treated uninfected DA and AO rats. Bar plot results showed that the main drivers of microbial variability were *NK4A214 group*, *UCG-005*, *Marvinbryantia*, *Christensenellaceae R-7 group*, *Allobaculum*, *Streptococcus*, *Eubacterium ventriosum group*, *Monoglobus*, and *Acetitomaculum*, which dominated in the AO group, while *Turicibacter* predominated in the DA group (Figure 5b).

### 3.2. Antibiotic-Induced Gut Dysbiosis Leads to Intestinal Inflammation

To investigate whether treatment with antibiotics affects immune homeostasis in uninfected rats, we examined basic immune response parameters in the lungs, gut, and peripheral blood (Table 3).

Although antibiotic treatment did not influence immune parameters in the lungs of uninfected AO rats, significantly higher levels of IL-1β and TNF were observed in the lungs of DA rats treated with antibiotics compared with water-treated DA rats. Examination of lung-draining mediastinal lymph nodes of uninfected DA and AO rats showed that antibiotics did not cause any changes in the expression levels of genes encoding cytokines responsible for the antifungal response.

In contrast to the minor immune changes in lung tissue, pronounced alterations in immune parameters were detected in the intestines of rats treated with antibiotics. Higher cecal mass was observed in rats of both strains treated with antibiotics. In DA rats, antibiotic treatment induced an increase in TNF content in the ileum and IL-17 in the ileum and cecum, while higher cytokine levels in the ileum (IL-17), cecum (IFN-γ), and colon (IL-1β, IL-6), and variable cytokine content in the duodenum (increased IFN-γ and decreased IL-10), were observed in antibiotic-treated AO rats compared with the respective water-treated controls. Regardless of antibiotic treatment, higher cytokine levels along different gut segments were found in AO rats compared with DA rats. In line with this, in both water- and antibiotic-treated rats, significantly higher IL-1β, IFN-γ, and IL-17 levels were noted in the duodenum, IL-17 in the ileum, and IL-10 in the colon of AO rats compared with DA rats. Among water-treated uninfected animals, higher levels of IL-10 in the duodenum and ileum and IL-1β in the cecum were noted in AO compared with DA rats, while among antibiotic-treated rats there were higher levels of IL-1β and IL-6 in the colon of AO rats compared with the respective DA rat group.

Oral consumption of antibiotics also affected mesenteric lymph nodes, increasing IFN-γ gene expression in both rat strains and increasing IL-17 gene expression in AO rats.

The only change in peripheral blood as a consequence of antibiotic consumption was an increase in the level of IL-6 in AO rat plasma.

In addition to immune parameters in lung and gut tissue homogenates, we also measured parameters of oxidative stress in the lungs and colon of animals treated with antibiotics. The only change observed was an increased content of reduced glutathione in the lungs of antibiotic-treated compared with water-treated DA rats (Table 4).

### 3.3. Administration of Antibiotics in DA and AO Rats Prior to Infection with A. fumigatus Causes More Pronounced Gut Dysbiosis but Not Lung Dysbiosis During Infection

To investigate whether rat strain-specific responses to antibiotic administration might cause rat strain-specific lung immune responses to the fungus *A. fumigatus*, water-treated and antibiotic-treated rats of both strains were intratracheally administered with *A. fumigatus*.

**Figure 6:**
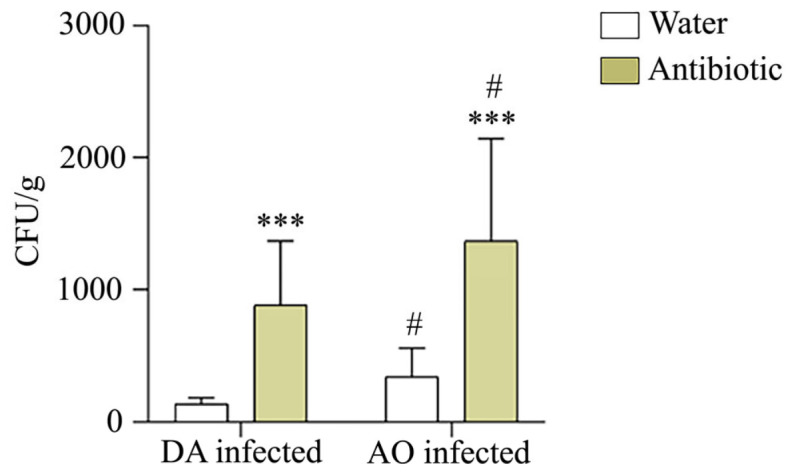
Effect of 30-day oral consumption of antibiotic solution on fungal burden in lung tissue of *A. fumigatus*-infected DA and AO rats. Results are expressed as mean ± SD. Statistically significant differences: *** *p* < 0.001 vs. water; # *p* < 0.05 vs. DA.

Assessment of fungal presence in the lungs on day 3 post-infection revealed slower elimination of this opportunistic pathogen from the lungs of both DA and AO antibiotic-treated groups (Figure 6). In addition, fungal elimination was slower in AO than in DA rats, regardless of water or antibiotic treatment.

**Figure 7:**
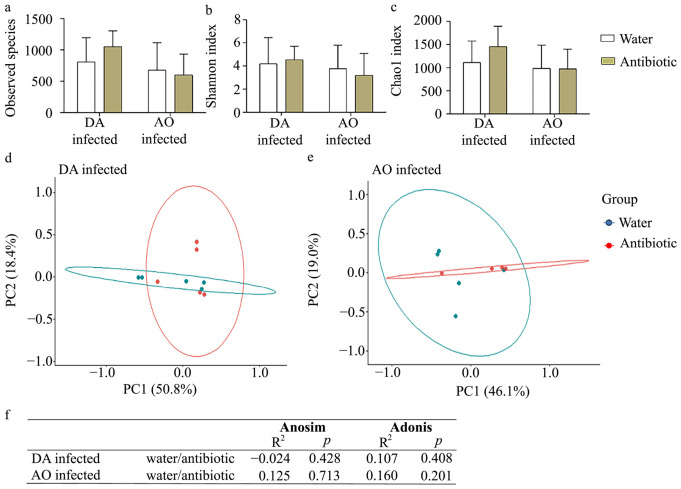
Effect of 30-day oral consumption of an antibiotic solution on lung bacterial microbiota in *A. fumigatus*-infected DA and AO rats: alpha diversity expressed through Observed species (**a**), Shannon index (**b**), and Chao1 index (**c**), and beta diversity expressed through Principal Coordinates Analysis (PCoA), based on Jensen–Shannon divergence at the genus level, in *A. fumigatus*-infected DA (**d**) and AO (**e**) rats. Differences in lung bacterial community in *A. fumigatus*-infected DA and AO rats, expressed through Adonis and ANOSIM, are depicted in (**f**). Results in (**a**–**c**) are presented as mean ± SD.

Antibiotic administration prior to infection with *A. fumigatus* did not cause changes in alpha diversity of lung microbiota in either DA or AO rats (Figure 7a–c), nor did it cause changes in the composition of the lung bacterial community in these strains (Figure 7d–f).

**Figure 8:**
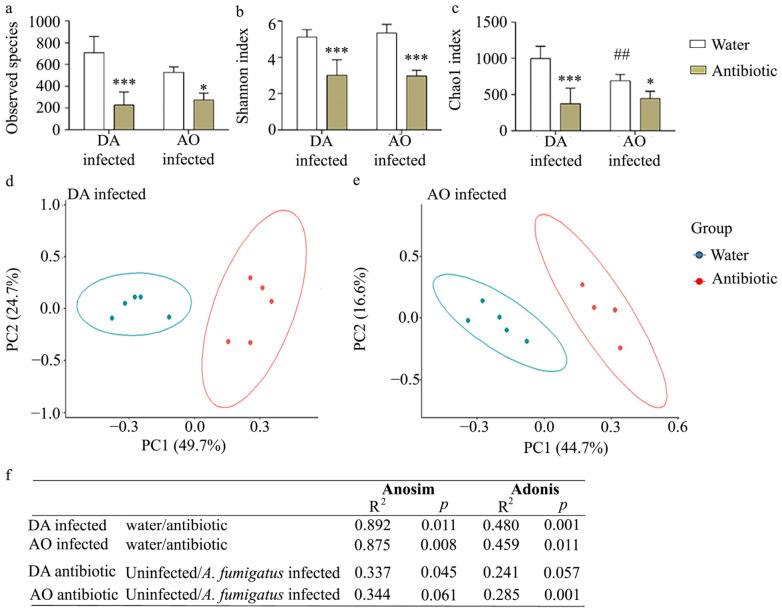
Effect of 30-day oral consumption of antibiotic solution on fecal bacterial microbiota in *A. fumigatus*-infected DA and AO rats: alpha diversity expressed through Observed species (**a**), Shannon index (**b**), and Chao1 index (**c**), and beta diversity expressed through Principal Coordinates Analysis (PCoA), based on Jensen–Shannon dissimilarity index at the genus level, in *A. fumigatus*-infected DA (**d**) and AO (**e**) rats. Differences in fecal bacterial community of *A. fumigatus*-infected DA and AO rats expressed through Adonis and ANOSIM are depicted in (**f**). Results in (**a**–**c**) are expressed as mean ± SD. Statistically significant differences: * *p* < 0.05 and *** *p* < 0.001 vs. water; ## *p* < 0.01 vs. DA.

However, antibiotic treatment prior to *A. fumigatus* infection affected microbial alpha diversity of the gut microbiota in both strains of *A. fumigatus*-infected rats, which was reflected as a decrease in the number of Observed species (Figure 8a), Shannon index (Figure 8b), and Chao1 index (Figure 8c). In both rat strains, the composition of the gut bacterial community in infected animals that received antibiotics was significantly different compared with infected animals that did not receive antibiotics (Figure 8d–f). Additionally, in both rat strains, gut microbiota analysis also showed significant differences between uninfected and *A. fumigatus*-infected animals that received antibiotics (Figure 8f).

**Figure 9:**
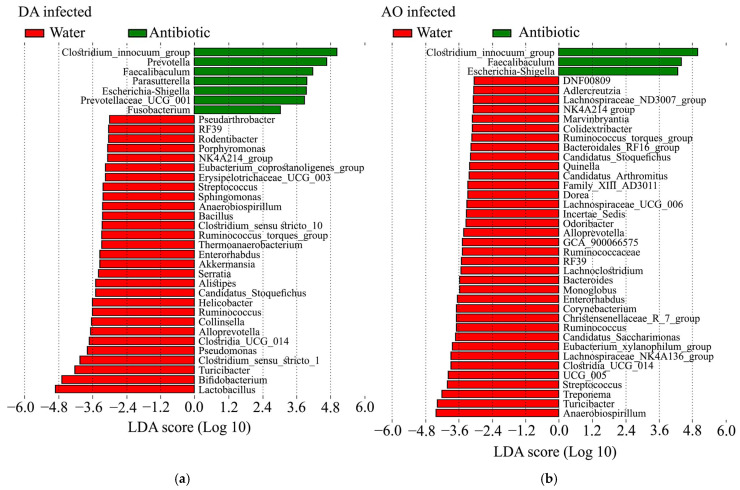
Effect of 30-day oral consumption of an antibiotic solution on gut bacterial biomarkers, presented through the Linear Discriminant Analysis Effect Size (LEfSe) method at the genus level, in *A. fumigatus*-infected DA (**a**) and AO (**b**) rats.

According to LEfSe analysis, a lower number of differentially abundant genera was detected in antibiotic-treated compared with water-treated infected animals of both DA and AO rat strains, indicating a selective pressure of antibiotic administration on the gut bacterial population following infection (Figure 9). Many genera repeatedly appear in both groups across strains. During pulmonary infection in water-treated groups of both DA and AO rats, we found consistent enrichment of the same genera (Figure 9). A similar pattern was also noted in antibiotic-treated groups (Figure 9), corroborating that these specific microbial signatures are created primarily by the treatments and not by the animal strain.

### 3.4. Antibiotic-Induced Gut Dysbiosis in A. fumigatus-Infected Rats Leads to Intestinal Inflammation

Since *A. fumigatus* infection in antibiotic-treated rats resulted in dysbiosis in the gut but not in the lungs, we investigated whether the relatively stable bacterial community in the lungs, as opposed to microbial alterations in the gut, affected immune response and oxidative stress parameters in these organs differently.

**Figure 10:**
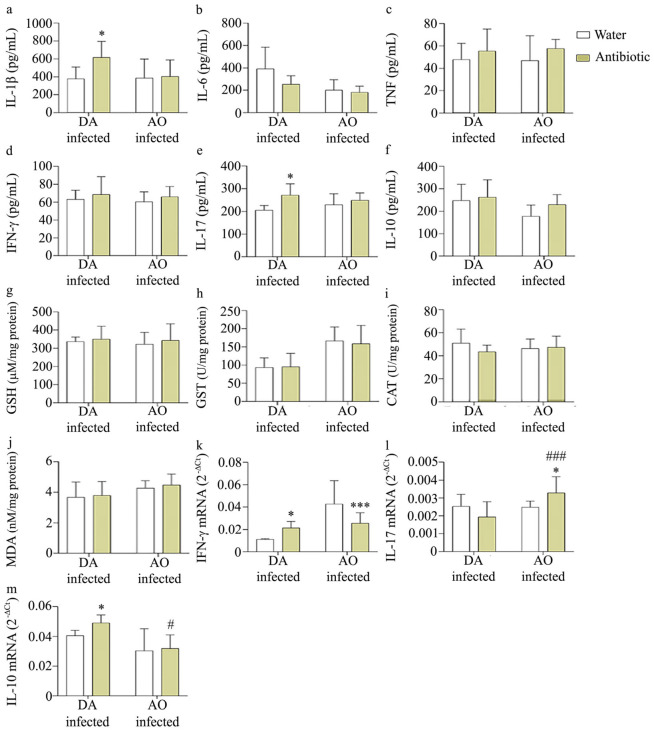
Effect of 30-day oral consumption of antibiotic solution in *A. fumigatus*-infected DA and AO rats on lung content of IL-1β (**a**), IL-6 (**b**), TNF (**c**), IFN-γ (**d**), IL-17 (**e**), and IL-10 (**f**); level of GSH (**g**), activity of GST (**h**) and CAT (**i**), and level of MDA (**j**) in the lungs; and the expression of genes for IFN-γ (**k**), IL-17 (**l**), and IL-10 (**m**) in the regional mediastinal lymph nodes. Results are expressed as mean ± SD. Statistically significant differences: * *p* < 0.05 and *** *p* < 0.001 vs. water; # *p* < 0.05 and ### *p* < 0.001 vs. DA.

Cytokine content analysis in the lungs during *A. fumigatus* infection showed significantly higher levels of IL-1β and IL-17 in DA animals treated with antibiotics compared with DA animals that received water (Figure 10a–f). In addition, in *A. fumigatus*-infected DA rats, higher lung MPO activity was noted in antibiotic-treated animals (0.78 ± 0.08 U/mL) compared with water-treated animals (0.54 ± 0.07 U/mL, *p* = 0.0005), while these changes were not detected in infected AO rats (0.79 ± 0.15 U/mL in antibiotic-treated vs. 0.67 ± 0.07 U/mL in water-treated AO rats, *p* = 0.129). However, there were no significant differences in lung levels of GSH and MDA or GST and CAT activity between antibiotic-treated and water-treated infected rats in either rat strain (Figure 10g–j). To examine whether antibiotic administration before *A. fumigatus* infection affects the activation of adaptive T cell responses to the fungus, we analyzed the expression levels of genes encoding cytokines of Th1 (IFN-γ), Th17 (IL-17), and Treg (IL-10) cells in regional mediastinal lymph nodes (Figure 10k–m). In DA rats, higher levels of IFN-γ and IL-10 gene expression were noted in antibiotic-treated compared with water-treated infected rats, while in AO rats, lower levels of IFN-γ and higher levels of IL-17 were detected in rats treated with antibiotics than in water-treated infected rats (Figure 10k–m).

**Figure 11:**
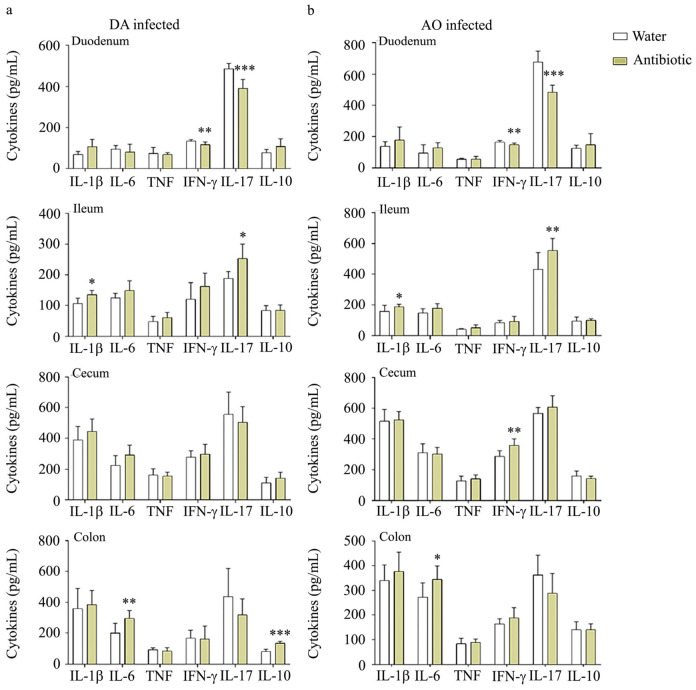
Effect of 30-day oral consumption of an antibiotic solution on the content of IL-1β, IL-6, TNF, IFN-γ, IL-17, and IL-10 in the duodenum, ileum, cecum, and colon of *A. fumigatus*-infected DA (**a**) and AO (**b**) rats. Results are expressed as mean ± SD. Statistically significant differences: * *p* < 0.05, ** *p* < 0.01, and *** *p* < 0.001 vs. water.

As previous results showed that dysbiosis in the gut coincided with gut inflammation, we assessed cytokine content in all segments of the gastrointestinal tract of infected animals that received antibiotics (Figure 11). Significantly lower levels of IFN-γ and IL-17 in the duodenum, and higher levels of IL-1β and IL-17 in the ileum and IL-6 in the colon, were observed in both DA and AO antibiotic-treated rats relative to water-treated infected rats. Additionally, a higher level of IL-10 in the colon of DA rats and a higher level of IFN-γ in the cecum of AO rats were noted as a consequence of antibiotic treatment of infected animals (Figure 11).

## 4. Discussion

The most common approach to investigating the role of gut microbiota in lung immune responses is the use of antibiotics characterized by systemic diffusion [25,28,29]. In this study, the antibiotics neomycin, gentamicin, and vancomycin were used because of their limited systemic absorption [47,48,49], thereby minimizing the direct effects of antibiotics on lung microbiota and immune responses and enabling a more straightforward assessment of gut microbiota influence on the lungs. This combination of antibiotics has a broad antibacterial spectrum, as vancomycin is effective against Gram-positive bacteria [50], while neomycin and gentamicin are primarily used to treat Gram-negative bacteria [48].

Oral administration of the antibiotic mix did not cause changes in the lung bacterial community in either rat strain, but it did cause gut dysbiosis in both DA and AO rats. Rat strain differences in antibiotic-induced gut microbiota dysbiosis, namely changes in gut microbiota composition without changes in richness and diversity (alpha diversity) in DA rats, as opposed to changes in gut microbiota composition associated with reduced diversity and a lower total number of bacterial species in AO rats, are unlikely to be caused by the differences in antibiotic dose consumed by the animals, because the daily intake was the same in both rat strains. Differential effects of antibiotic treatment were probably due to variability in basal gut microbiota composition between DA and AO rats. Variation in microbiota response to the same antibiotics has been noted in the human population [44] and confirmed in mice colonized with human microbiota from different donors [51].

Differences in gut microbiota between healthy DA and AO rats persisted after antibiotic treatment. Administration of the antibiotic mix in DA rats caused an increase in the relative abundance of members of the class Gammaproteobacteria (representatives of the phylum Proteobacteria). An increase in this class was also noted in the gut of mice orally treated with different mixtures of antibiotics (ampicillin, gentamicin, metronidazole, neomycin, and vancomycin) [52,53]. Although present at low abundance in the mammalian gut, a higher level of Proteobacteria is associated with numerous pathological conditions and can even be observed as a unique marker of microbial dysbiosis and a factor contributing to disease [54], such as metabolic disorders [55] and gut inflammation [56]. The increased abundance of bacteria from this class in DA rats may be driven by the presence of an antibiotic resistance gene (ARG), because these taxa are the major carriers of ARG [54,57]. Additionally, representatives of the phylum Proteobacteria produce some of the most potent pro-inflammatory lipopolysaccharides (LPS) [58], which can activate different immune cells and elicit a strong pro-inflammatory response [59]. These findings support the hypothesis that, in the absence of any change in lung microbiota, the increased abundance of LPS-producing bacterial species from the Proteobacteria phylum (Gammaproteobacteria) in the gut of DA rats may be responsible for antibiotic-induced disruption of pulmonary immune homeostasis, which is seen in DA but not in AO rats. Bacterial endotoxins (LPS) from the gut can enter the circulation and reach the lungs via the bloodstream, providing an additional basis for the gut–lung axis [60]. On the other hand, diminished relative abundance of Coriobacteriia, which was found in antibiotic-treated AO rats, has been reported in children with inflammatory bowel disease (IBD) [61], in patients with rheumatoid arthritis [62], and in an inflammation-induced model of depression [63], whereas higher abundance has been detected in adults with IBD [64] compared with healthy controls. This clear discrepancy, as well as the fact that Coriobacteriia are important pathobionts associated with numerous pathologies [65], confirms that an increase or decrease in the abundance of a single bacterial class or species following antibiotic treatment cannot, by itself, explain the overall complex influence of antibiotic-induced gut dysbiosis on immune changes.

Rat strain-related differences in response to the antibiotics alone, or to the combined effect of antibiotics and infection, were evident according to the LEfSe analysis. Although the LEfSe analysis showed strain-related differences in the abundance of genera between the examined groups, some genera appear to show a robust pattern in response to antibiotics, irrespective of rat strain. In that context, in both DA and AO rat strains, antibiotic administration induced a higher abundance of the *Clostridium innocuum group*, which could be attributed to intrinsic resistance to vancomycin [66]. The enrichment of *Peptostreptococcus* as a common feature in both rat strains has also been implicated in chronic inflammatory gut disorders, including ulcerative colitis and IBD [67,68].

It also appears that antibiotic treatment in AO rats caused a more pathogenic gut microbiota profile characterized by the enrichment of disease- and gut inflammation-associated bacteria such as *Enterococcus*, *Escherichia-Shigella*, and *Fusobacterium* [69,70,71], unlike in antibiotic-treated DA animals. Although the administration of antibiotics was associated with the enrichment of *Escherichia-Shigella* in both rat strains, it was dependent on both host genetic and treatment-related factors. While a higher abundance of this genus was noted in AO rats following antibiotic treatment, it appears that, in DA rats, antibiotic administration created a favorable environment for *Escherichia-Shigella*, but it was infection with *A. fumigatus* that triggered this enrichment.

Antibiotic-induced dysbiosis was accompanied by disturbances in gut immune homeostasis in both rat strains. The higher relative cecal mass observed in both strains is likely caused by lower bacterial diversity in the gut, as has also been reported in germ-free or reduced-microbiota mice [52]. Moreover, increased cecal mass could also be a consequence of vancomycin treatment [72]. In addition, antibiotic-induced intestinal inflammation was limited to the ileum and cecum in DA rats, whereas in AO rats, it was detected in all segments of the gastrointestinal tract, probably as a result of more pronounced dysbiosis in AO compared with DA rats. Gut dysbiosis induces intestinal inflammation [73,74,75,76], and gut dysbiosis and inflammation can disrupt intestinal barrier integrity and increase its permeability, which can lead to pathogen translocation via blood circulation into distant organs [77,78,79]. In that context, stronger dysbiotic changes and inflammation in all gut segments in AO rats most likely compromised gut integrity, causing higher levels of IL-6 in the blood plasma of these animals.

Administration of antibiotics before infection with the fungus *A. fumigatus* led to a higher conidial burden in both DA and AO rats compared with their water-treated counterparts, but this effect was more pronounced in AO rats. Greater susceptibility to infections (slower clearance of pathogens from the lungs) was also observed in animals treated with antibiotics before infection with the bacteria *Pseudomonas aeruginosa* [26,80], *Streptococcus pneumoniae*, and *Klebsiella pneumoniae* [24,27]. The increased level of infection, measured by CFU in animals given antibiotics, was probably not the result of overall immune suppression in the lungs, because comparable cytokine levels were noted in AO water- and antibiotic-treated animals. In contrast to the AO strain, even higher levels of IL-1β, IL-17, and MPO were detected in the lungs of antibiotic-treated infected animals compared with water-treated infected animals of DA rat strain. Higher cytokine levels were also noted in antibiotic-treated animals infected with the bacteria *E. coli* or *S. pneumoniae* [24,25,27]. It was noted that treatment with antibiotics could lead to reduced macrophage activity [24,25,27], which would impair fungal clearance, and gentamicin and streptomycin were shown to significantly inhibit macrophage activation at concentrations higher than 40 mg/L [81]. Administration of antibiotics also affected activation of the adaptive immune response in regional lymph nodes in response to *A. fumigatus* infection, by increasing Th1 (production of IFN-γ), and Treg cell (production of IL-10) activity in DA animals, while increasing Th17 (production of IL-17) but reducing Th1 cell activity in AO rats. Given that Th1 and Th17 cells confer antifungal activity by producing IFN-γ and IL-17 [82], and that IL-10 has a largely detrimental role during fungal disease [83], higher levels of anti-inflammatory IL-10 and lower levels of pro-inflammatory IFN-γ in antibiotic-treated DA and AO animals, respectively, probably caused an inadequate antifungal response compared with animals of both strains treated with water.

Antibiotic treatment *per se* induced lung inflammation in DA rats by increasing the production of IL-1β and TNF. Considering that IL-17 recruits neutrophils to the airways [84], its increase in lung tissue following *A. fumigatus* infection of DA rats probably resulted in higher neutrophil numbers in the lungs. This, in turn, led to increased MPO activity in the already inflammatory microenvironment of DA rat lung tissue, along with additional lung tissue IL-1β production. Mechanistically, lung inflammation in DA, but not in AO rats, may result from the inflammatory effect of LPS released by representatives of the phylum Proteobacteria, which were enriched in the gut tissue of DA rats following antibiotic treatment, as previously discussed. Subtle differences in the integrity of the colonic epithelial cell lining between DA and AO rats were previously suggested [85], but these assumptions remain speculative in the absence of experimental confirmation. In contrast, there was no change in lung cytokine production during antibiotic treatment of AO rats, nor was it affected by *A. fumigatus* infection, so IL-17 produced by mediastinal lymph nodes in the absence of an increase in IFN-γ probably failed to facilitate neutrophil influx into lung tissue and more efficiently eliminate the fungus. It must be emphasized that changes in susceptibility to infection caused by the fungus *A. fumigatus* were not the result of disrupted lung microbiota, because the antibiotics did not cause dysbiosis in this organ, but were induced by antibiotic-induced gut dysbiosis in a rat strain-specific manner.

It has previously been shown that infection with *A. fumigatus* induces gut inflammation in both rat strains, which was associated with gut dysbiosis only in rats of the DA strain [22]. Hence, in the present study, disturbances in gut community composition in infected DA and AO rat strains were probably caused by the combined effect of antibiotics and gut inflammation, the latter being caused by the lung fungal infection.

To explain the variability in gut microbiota responses to the same antibiotics in DA and AO rats, which may have further shaped their responses to pulmonary fungal infection, we clustered the bacterial community based on three discriminative genera. Since the definition of enterotype [86], numerous studies have shown enterotype-dependent variability in responses to different interventions, such as diet [87], probiotics [88], or antibiotic administration [51]. However, later analyses indicated that samples could not be easily separated into distinct clusters, suggesting a continuous gradient of microbiota ranging from a *Bacteroides*-driven to a *Prevotella*-driven type rather than discrete enterotypes [89,90]. Since the Silhouette score (a clustering metric) of 0.29 suggested overlapping clusters and not distinctive enterotypes, we analyzed whether the microbiota of our rat strains might follow a continuous gradient based on *Bacteroides/Prevotella* abundance. The analysis showed clear cluster separation. Although these genera were inversely related, with higher *Bacteroides* in DA rats and higher *Prevotella* abundance in AO rats, these taxa were not the main drivers of community variation, according to a bar plot showing the genera responsible for the observed differences in gut microbiota community. Although we did not find *Bacteroides/Prevotella* to be the strongest determinants in DA and AO rats, that does not indicate their biological irrelevance. These taxa can play an important role in host ecological structure and metabolic profile, serving as both markers of metabolic disease risk and therapeutic targets [91].

Enterotype-driven responses to antibiotic treatment have been confirmed in germ-free mice humanized with microbiota from donors with the *Bacteroides* and *Prevotella* enterotypes, respectively. While the *Prevotella* enterotype underwent a significant alteration following antibiotic administration, the *Bacteroides* enterotype remained relatively stable [51]. As DA rats showed unchanged gut microbial richness following antibiotic treatment, in contrast to the significantly reduced microbiota diversity in AO rats, it may be suggested that *Bacteroides* may have contributed to this adaptive response to antibiotic pressure. In that context, not only do *Bacteroides* possess inherited or acquired antimicrobial resistance [92], but these bacteria also show high genetic plasticity and can incorporate genes from other bacteria [93,94]. These traits likely facilitate adaptation to environmental stressors within the gut. Although they are mostly beneficial, *Bacteroides* can also indirectly support the growth of harmful bacteria [91]. As antibiotic administration disrupts the gut microbiota and reduces microbial competition, it creates permissive conditions for pathogens to better access nutrients provided by *Bacteroides* [95]. Regarding the *Prevotella* enterotype, Arumugam and colleagues [86] reported a subpopulation of bacteria within this cluster, such as *Streptococcus*, which was noted in our study (this genus dominated in the AO uninfected water-treated group). The same group of authors noted co-occurrence of *Escherichia/Shigella*, also observed in AO rats after antibiotic administration. Considering that some members of *Streptococcus* [96] and the *Escherichia/Shigella* group [97] are recognized as markers of systemic inflammation, and that AO rats have a specific microbial signature characterized by enrichment of *Streptococcus* in the basal state followed by enrichment of *Escherichia/Shigella* after antibiotic administration, it could be suggested that these taxa may have potentially contributed to the systemic inflammatory response associated with elevated IL-6 following antibiotic treatment in AO rats.

The use of three different antibiotics in the present study does not preclude the notion that different bacterial taxa display different antibiotic resilience due to intrinsic resistance mechanisms and recovery capacity [98]. Finally, even though the prevalence of *Bacteroides* or *Prevotella* gut communities has been shown to be driven by long-term dietary patterns, with *Bacteroides* associated with complex carbohydrate consumption and *Prevotella* with an animal protein- and saturated fat-rich diet [99,100], animals in our study were housed in the same animal facility and received the same diet, so these variations seem to be genotype-driven rather than the result of environmental factors.

While data on the relationship between enterotypes and disease remain limited, some studies have suggested an association of certain enterotypes not only with gut disorders but also with the development of diseases in distant organs [101,102]. Given the complex nature of the gut microbiota and the intricate influence of gut microbial community variation on immune changes, enterotype alone is unlikely to be a prognostic biomarker of any disease [89], but it may certainly contribute to disease onset and progression.

## 5. Conclusions

Overall, our results suggest that rat strain-specific enterotypes shape the strain-specific response to antibiotic treatment. Even though antibiotics induced gut dysbiosis in both DA and AO rats and did not have effect on lung microbial community in either of the strains, gut inflammation brought about by antibiotics in both rat strains was paralleled by altered cytokine profile in lung tissue only in rats of DA strain. Moreover, antibiotic-induced gut dysbiosis was associated with less efficient clearance of *A. fumigatus* from the lungs in both strains, with clearance remaining more efficient in DA rats.

The use of an antibiotic mixture with poor systemic absorption supports the involvement of the gut–lung axis in modulating the host antifungal response. Moreover, by using two rat strains with a different continuous gradient of microbiota, ranging from a *Bacteroides*-driven to a *Prevotella*-driven type, our study highlights the importance of the interplay of genotype-specific host gut microbial community with the environmental constraints in shaping individual immune makeup.

It is important to note that although some reports in rodents have described enterotype-like community structures that may be similar to those in human populations [87,103], gut clustering studies in rats are still limited. Considering the differences in the *Bacteroides/Prevotella* proportion between our rat strains, we did not want to overlook the potential contribution of this ratio to strain-specific responses. Given the relatively small gut sample size, these data should be interpreted with caution, as they may limit a more accurate assessment of the contribution of these taxa to microbiota clustering. Moreover, we believe that, despite the absence of a negative control during lung tissue sequencing, these observations can serve as preliminary findings on how *Bacteroides* and *Prevotella* may be associated with individual variations in lung responses to different factors. Additionally, the consistent significant differences we confirmed in microbial and immunological analyses between the DA and AO rat strains across multiple analytical approaches may strengthen confidence in our findings.

## Figures and Tables

**Table 1:** Primers used to detect gene expression in mediastinal and mesenteric lymph node cells.

	5′–3′ Forward	5′–3′ Reverse
β-actin	CCCTGGCTCCTAGCACCAT	GAGCCACCAATCCACACAGA
IFN-γ	TGGCATAGATGTGGAAGAAAAGAG	TGCAGGATTTTCATGTCACCA
IL-17	ATCAGGACGCGCAAACATG	TGATCGCTGCTGCCTTCAC
IL-10	GAAGACCCTCTGGATACAGCTGC	TGCTCCACTGCCTTGCTTTT

**Table 2:** Effect of 30-day oral consumption of an antibiotic solution on daily fluid and antibiotic intake in uninfected DA and AO rats.

	DA Uninfected		AO Uninfected	
	Water	Antibiotics	Water	Antibiotics
Daily fluid intake (mL)	31.4 ± 2.6	38.0 ± 3.1 ***	42.9 ± 5.1 ***	46.3 ± 3.1 * ^###^
Daily Antibiotic intake (g/kg BM)				
Neomycin	/	0.11 ± 0.02	/	0.10 ± 0.02
Gentamicin	/	0.11 ± 0.02	/	0.10 ± 0.02
Vancomycin	/	0.05 ± 0.01	/	0.05 ± 0.01

Results are expressed as mean ± SD. Statistically significant differences: * *p* < 0.05 and *** *p* < 0.001 vs. water; ^###^ *p* < 0.001 vs. DA.

**Table 3:** Effect of 30-day oral consumption of antibiotic solution on immune homeostasis in the lung, gut, and plasma of uninfected DA and AO rats.

Examined Parameters	DA		AO	
	Water	Antibiotics	Water	Antibiotics
**Lungs**				
Relative lung mass (g)	0.67 ± 0.06	0.69 ± 0.08	0.64 ± 0.14	0.58 ± 0.09
Cytokine level (pg/mL)				
IL-1β	368.4 ± 154.9	643.8 ± 203.1 *	595.7 ± 184.6 ^#^	559.3 ± 171.7
IL-6	478.8 ± 93.8	383.6 ± 143.0	326.3 ± 177.0	283.3 ± 140.1
TNF	58.9 ± 6.6	87.7 ± 21.3 **	62.5 ± 12.9	73.7 ± 17.4
IFN-γ	37.0 ± 5.8	40.3 ± 11.0	48.3 ± 9.6	44.0 ± 16.1
IL-17	175.3 ± 32.7	144.4 ± 24.2	176.2 ± 58.3	172.2 ± 27.8
IL-10	291.5 ± 75.2	272.1 ± 71.0	251.0 ± 89.2	245.0 ± 95.0
MPO activity (U/mL)	0.47 ± 0.08	0.57 ± 0.13	0.32 ± 0.09	0.40 ± 0.09
Regional mediastinal lymph nodes				
Number of cells (×10^6^)	13.5 ± 7.2	23.3 ± 14.0	12.8 ± 5.0	14.7 ± 8.0
IFN-γ (2^−ΔCt^)	0.008 ± 0.001	0.012 ± 0.002	0.009 ± 0.004	0.012 ± 0.002
IL-17 (2^−ΔCt^)	0.002 ± 0.000	0.002 ± 0.000	0.001 ± 0.000	0.001 ± 0.000
IL-10 (2^−ΔCt^)	0.033 ± 0.005	0.038 ± 0.004	0.030 ± 0.006	0.019 ± 0.004
**Gut**				
Relative cecal mass	1.74 ± 0.28	5.43 ± 1.74 ***	1.82 ± 0.54	5.53 ± 1.37 ***
Cytokine level (pg/mL)				
Duodenum				
IL-1β	63.0 ± 15.9	62.5 ± 37.2	159.6 ± 37.6 ^###^	178.3 ± 43.6 ^###^
IL-6	75.0 ± 26.5	101.4 ± 13.3	92.0 ± 17.4	94.0 ± 40.6
TNF	59.7 ± 15.2	65.8 ± 18.5	65.6 ± 13.8	69.7 ± 12.8
IFN-γ	118.0 ± 10.9	128.1 ± 16.4	137.3 ± 6.5 ^##^	156.7 ± 11.0 ** ^##^
IL-17	414.8 ± 41.5	408.3 ± 55.4	501.6 ± 45.1 ^##^	496.3 ± 50.5 ^##^
IL-10	65.2 ± 13.5	94.0 ± 29.7	143.8 ± 60.8 ^###^	93.8 ± 33.7 *
Ileum				
IL-1β	120.0 ± 20.5	103.0 ± 17.4	160.8 ± 24.6	144.0 ± 81.1
IL-6	136.2 ± 18.8	132.1 ± 57.8	169.0 ± 31.7	167.0 ± 69.3
TNF	38.3 ± 12.7	65.8 ± 27.6 *	47.1 ± 22.8	48.3 ± 17.1
IFN-γ	100.0 ± 15.4	136.5 ± 54.4	105.6 ± 23.0	100.0 ± 16.7
IL-17	118.5 ± 21.2	310.2 ± 96.0 ***	297.3 ± 39.7 ^###^	511.1 ± 107.2 *** ^###^
IL-10	75.0 ± 20.9	74.7 ± 19.5	106.6 ± 18.3 ^#^	95.2 ± 29.7
Cecum				
IL-1β	396.7 ± 48.7	415.8 ± 80.7	490.0 ± 36.3 ^##^	447.5 ± 38.3
IL-6	250.0 ± 33.0	225.8 ± 42.2	286.9 ± 47.5	287.9 ± 38.5 ^##^
TNF	121.8 ± 23.7	126.7 ± 7.7	132.8 ± 35.3	147.1 ± 28.5
IFN-γ	292.9 ± 36.6	323.8 ± 36.3	266.7 ± 23.6	364.3 ± 50.7 ***
IL-17	348.1 ± 190.1	562.4 ± 84.5 **	470.4 ± 27.3	573.5 ± 108.2
IL-10	120.0 ± 25.2	151.8 ± 21.8	151.7 ± 15.5	184.4 ± 44.6
Colon				
IL-1β	235.0 ± 31.5	240.0 ± 58.5	273.1 ± 48.9	340.8 ± 70.4 * ^##^
IL-6	205.6 ± 53.1	247.5 ± 71.4	227.5 ± 108.5	375.0 ± 101.6 ** ^#^
TNF	73.3 ± 17.8	82.7 ± 16.5	93.3 ± 12.6	91.1 ± 24.4
IFN-γ	128.6 ± 38.5	169.4 ± 46.6	183.3 ± 71.3	197.2 ± 54.5
IL-17	270.9 ± 67.5	290.1 ± 82.0	286.0 ± 91.5	333.3 ± 53.3
IL-10	93.3 ± 12.9	105.5 ± 15.3	146.6 ± 21.9 ^###^	165.0 ± 26.9 ^###^
Mesenteric lymph nodes				
Number of cells (×10^6^)	54.4 ± 20.4	43.9 ± 11.3	70.8 ± 11.5	63.2 ± 23.7
IFN-γ (2^−ΔCt^)	0.005 ± 0.002	0.018 ± 0.003 ***	0.004 ± 0.001	0.007 ± 0.001 * ^###^
IL-17 (2^−ΔCt^)	0.002 ± 0.000	0.002 ± 0.000	0.001 ± 0.000 ^##^	0.002 ± 0.000 ***
IL-10 (2^−ΔCt^)	0.018 ± 0.008	0.018 ± 0.007	0.018 ± 0.007	0.018 ± 0.001
**Peripheral blood**				
Leukocytes (×10^9^/L)	5.8 ± 1.9	4.7 ± 2.0	3.0 ± 1.2	3.1 ± 0.5
Lymphocytes (×10^9^/L)	49.0 ± 13.1	61.2 ± 7.4	35.5 ± 5.5	42.1 ± 14.6
Neutrophils (×10^9^/L)	48.0 ± 12.8	36.1 ± 7.2	60.1 ± 5.3	53.4 ± 14.6
Monocytes (×10^9^/L)	1.5 ± 0.3	1.2 ± 0.3	1.6 ± 0.4	1.7 ± 0.5
Eosinophils (×10^9^/L)	1.3 ± 0.5	1.4 ± 0.4	2.0 ± 0.7	2.3 ± 0.8
Basophils (×10^9^/L)	0.4 ± 0.1	0.3 ± 0.2	0.3 ± 0.1	0.4 ± 0.1
Erythrocytes (×10^12^/L)	8.2 ± 0.3	8.2 ± 0.2	8.2 ± 0.6	8.5 ± 0.8
Hemoglobin (g/L)	147.6 ± 5.0	147.0 ± 3.9	151.6 ± 4.7	152.5 ± 6.5
Hematocrit (%)	0.45 ± 0.01	0.44 ± 0.02	0.46 ± 0.02	0.48 ± 0.02
Thrombocytes (×10^9^/L)	782.8 ± 35.1	753.2 ± 19.9	857.6 ± 26.0	802.5 ± 66.2
Plasma cytokines				
IL-6 (pg/mL)	34.3 ± 10.6	43.6 ± 16.4	35.0 ± 18.7	56.7 ± 11.2 *
TNF (pg/mL)	58.6 ± 7.9	54.2 ± 13.1	50.0 ± 21.9	49.0 ± 16.8
IL-17 (pg/mL)	108.0 ± 24.9	135.8 ± 16.2	108.0 ± 23.0	126.5 ± 27.0

Results are expressed as mean ± SD. Statistically significant differences: * *p* < 0.05, ** *p* < 0.01 and *** *p* < 0.001 vs. water; ^#^ *p* < 0.05, ^##^ *p* < 0.01 and ^###^ *p* < 0.001 vs. DA.

**Table 4:** Effect of 30-day oral consumption of an antibiotic solution on parameters of oxidative stress and antioxidative defense in the lungs and colon of uninfected DA and AO rats.

Examined Parameters	DA		AO	
	Water	Antibiotics	Water	Antibiotics
**Lungs**				
GSH (μM/mg proteins)	309.6 ± 7.8	397.0 ± 19.3 *	311.8 ± 22.8	359.1 ± 33.5
GST (U/mg proteins)	114.2 ± 9.8	117.2 ± 5.8	170.7 ± 14.4	182.5 ± 6.8
CAT (U/mg proteins)	53.0 ± 8.6	53.8 ± 7.8	50.3 ± 12.4	48.1 ± 9.4
MDA (nM/mg proteins)	3.9 ± 0.4	3.6 ± 0.1	3.9 ± 0.3	3.8 ± 0.3
**Colon**				
GSH (μM/mg proteins)	85.0 ± 23.3	84.0 ± 20.3	128.5 ± 25.7	146.6 ± 31.9
GST (U/mg proteins)	35.6 ± 4.6	35.1 ± 4.7	47.8 ± 6.5	36.2 ± 4.3
CAT (U/mg proteins)	22.3 ± 1.7	26.6 ± 2.5	30.6 ± 3.1	24.1 ± 2.4
MDA (nM/mg proteins)	5.9 ± 0.6	5.5 ± 0.5	5.8 ± 0.6	5.0 ± 0.4

Results are expressed as mean ± SD. Statistically significant differences: * *p* < 0.05 vs. water.

## Data Availability

The raw 16S rRNA gene sequence data reported in this study have been deposited in the European Nucleotide Archive (https://www.ebi.ac.uk/ena. accessed on: 1 June 2026.) under study accession number PRJEB113786. The other raw data supporting the conclusions of this article will be made available by the authors on request.

## Supplementary Materials

The following supporting information can be downloaded at: https://www.mdpi.com/article/10.3390/biology15141132/s1, Supplementary File S1: Data on Reads obtained per sample (Table S1), Quality control data for FASTQ files (Table S2), and The primer sequences table (Table S3); Supplementary File S2: Daily fluid and antibiotic intake in uninfected DA and AO rats (Table S1); Alpha diversity in the lungs and feces of uninfected DA and AO rats (Table S2); Alpha diversity in the lungs and feces of infected DA and AO rats (Table S3); The effect of antibiotic treatment on the relative abundance of fecal bacteria at the class level in uninfected DA and AO rats (Table S4); Top 10 drivers of community variations in uninfected DA and AO rats (Table S5); Immune homeostasis and oxidative parame-ters in the lungs, gut, and plasma of uninfected DA and AO rats (Table S6); The effect of antibiotic treatment on the fungal burden in the lungs of infected DA and AO rats (Table S7); and Immune homeostasis and oxidative parameters in the lungs, gut, and plasma of infected DA and AO rats (Table S8).

## Abbreviations

The following abbreviations are used in this manuscript:

*A. fumigatus*	*Aspergillus fumigatus*
ANOSIM	analysis of similarities
ANOVA	analysis of variance
AO	Albino Oxford rat strain
ARG	antibiotic resistance gene
B/P	Bacteroides/Prevotella ratio
BM	body mass
b.w.	body weight
BSA	bovine serum albumin
CAT	catalase
cDNA	complementary DNA
CFU	colony-forming units
ΔCt	delta threshold cycle
DA	Dark Agouti rat strain
DTNB	5,5-dithio-bis-(2-nitrobenzoic acid)
*E. coli*	*Escherichia coli*
EDTA	ethylenediaminetetraacetic acid
ELISA	enzyme-linked immunosorbent assay
FDR	false discovery rate
GSH	reduced glutathione
GST	glutathione S-transferase
IBD	inflammatory bowel disease
IFN	interferon
IL	interleukin
LDA	linear discriminant analysis
LEfSe	linear discriminant analysis effect size
LPS	lipopolysaccharide
MDA	malondialdehyde
MMLV	Moloney murine leukemia virus
MPO	myeloperoxidase
OTU	operational taxonomic unit
PAM	partitioning around medoids
PC1	first principal coordinate
PCoA	Principal Coordinates Analysis
PMSF	phenylmethanesulfonyl fluoride
PTB	pulmonary tuberculosis
RT-PCR	Reverse Transcription Real-Time Polymerase Chain Reaction
*S. pneumoniae*	*Streptococcus pneumoniae*
SMA	Sabouraud maltose agar
TBA	2-thiobarbituric acid
TCA	trichloroacetic acid
Th1	T helper 1 cells
Th17	T helper 17 cells
TNF	tumor necrosis factor
Treg	regulatory T cells

## References

[1] Hillman E.T., Lu H., Yao T., Nakatsu C.H. (2017). Microbial ecology along the gastrointestinal tract. Microbes Environ..

[2] Afzaal M., Saeed F., Shah Y.A., Hussain M., Rabail R., Socol C.T., Hassoun A., Pateiro M., Lorenzo J.M., Rusu A.V. (2022). Human gut microbiota in health and disease: Unveiling the relationship. Front. Microbiol..

[3] Yu X., Yu X., Wang Y., Guo X., Wang C., Wang F. (2025). Respiratory diseases and the gut microbiota: An updated review. Front. Cell. Infect. Microbiol..

[4] Lin J., Chen D., Yan Y., Pi J., Xu J., Chen L., Zheng B. (2024). Gut microbiota: A crucial player in the combat against tuberculosis. Front. Immunol..

[5] Frati F., Salvatori C., Incorvaia C., Bellucci A., Di Cara G., Marcucci F., Esposito S. (2018). The role of the microbiome in asthma: The gut–lung axis. Int. J. Mol. Sci..

[6] McAleer J.P., Kolls J.K. (2018). Contributions of the intestinal microbiome in lung immunity. Eur. J. Immunol..

[7] Sokolowska M., Frei R., Lunjani N., Akdis C.A., O’Mahony L. (2018). Microbiome and asthma. Asthma Res. Pract..

[8] Vaughan A., Frazer Z.A., Hansbro P.M., Yang I.A. (2019). COPD and the gut-lung axis: The therapeutic potential of fibre. J. Thorac. Dis..

[9] Thavamani A., Salem I., Sferra T.J., Sankararaman S. (2021). Impact of altered gut microbiota and its metabolites in cystic fibrosis. Metabolites.

[10] Dilantika C., Sedyaningsih E.R., Kasper M.R., Agtini M., Listiyaningsih E., Uyeki T.M., Burgess T.H., Blair P.J., Putnam S.D. (2010). Influenza virus infection among pediatric patients reporting diarrhea and influenza-like illness. BMC Infect. Dis..

[11] Qin N., Zheng B., Yao J., Guo L., Zuo J., Wu L., Zhou J., Liu L., Guo J., Ni S. (2015). Influence of H7N9 virus infection and associated treatment on human gut microbiota. Sci. Rep..

[12] Chai Y., Li M., Deng X., Ma C., Zhou N., Chen Y., Yao Y., Li K., Gong W., Lei H. (2025). Gut microbiota and tuberculosis infection: Interaction and therapeutic potential. Gut Microbes.

[13] Li W., Zhu Y., Liao Q., Wang Z., Wan C. (2019). Characterization of gut microbiota in children with pulmonary tuberculosis. BMC Pediatr..

[14] Han M., Wang X., Zhang J., Su L., Ishaq H.M., Li D., Cui J., Zhao H., Yang F. (2024). Gut bacterial and fungal dysbiosis in tuberculosis patients. BMC Microbiol..

[15] Ye S., Wang L., Li S., Ding Q., Wang Y., Wan X., Ji X., Lou Y., Li X. (2022). The correlation between dysfunctional intestinal flora and pathology feature of patients with pulmonary tuberculosis. Front. Cell Infect. Microbiol..

[16] Wang S., Yang L., Hu H., Lv L., Ji Z., Zhao Y., Zhang H., Xu M., Fang R., Zheng L. (2022). Characteristic gut microbiota and metabolic changes in patients with pulmonary tuberculosis. Microb. Biotechnol..

[17] Groves H.T., Cuthbertson L., James P., Moffatt M.F., Cox M.J., Tregoning J.S. (2018). Respiratory disease following viral lung infection alters the murine gut microbiota. Front. Immunol..

[18] Gu S., Chen Y., Wu Z., Chen Y., Gao H., Lv L., Guo F., Zhang X., Luo R., Huang C. (2020). Alterations of the gut microbiota in patients with Coronavirus disease 2019 or H1N1 influenza. Clin. Infect. Dis..

[19] Zuo T., Zhang F., Lui G.C.Y., Yeoh Y.K., Li A.Y.L., Zhan H., Wan Y., Chung A.C.K., Cheung C.P., Chen N. (2020). Alterations in gut microbiota of patients with COVID-19 during time of hospitalization. Gastroenterology.

[20] Fan J., Liu S., Zhang H., Jin C., Wu N. (2026). Dysbiosis of the gut–lung axis and its immune correlates during pulmonary *Cryptococcus neoformans* infection. J. Fungi.

[21] Kulas J., Mirkov I., Tucovic D., Zolotarevski L., Glamoclija J., Veljovic K., Tolinacki M., Golic N., Kataranovski M. (2019). Pulmonary Aspergillus fumigatus infection in rats affects gastrointestinal homeostasis. Immunobiology.

[22] Popovic D., Kulas J., Tucovic D., Popov Aleksandrov A., Malesevic A., Glamoclija J., Brdaric E., Sokovic Bajic S., Golic N., Mirkov I. (2023). Gut microbial dysbiosis occurring during pulmonary fungal infection in rats is linked to inflammation and depends on healthy microbiota composition. Microbiol. Spectr..

[23] Dumas A., Bernard L., Poquet Y., Lugo-Villarino G., Neyrolles O. (2018). The role of the lung microbiota and the gut–lung axis in respiratory infectious diseases. Cell Microbiol..

[24] Schuijt T.J., Lankelma J.M., Scicluna B.P., de Sousa e Melo F., Roelofs J.J., de Boer J.D., Hoogendijk A.J., de Beer R., de Vos A., Belzer C. (2016). The gut microbiota plays a protective role in the host defence against pneumococcal pneumonia. Gut.

[25] Chen L.W., Chen P.H., Hsu C.M. (2011). Commensal microflora contribute to host defense against Escherichia coli pneumonia through Toll-like receptors. Shock.

[26] Robak O.H., Heimesaat M.M., Kruglov A.A., Prepens S., Ninnemann J., Gutbier B., Reppe K., Hochrein H., Suter M., Kirchning J.C. (2018). Antibiotic treatment–induced secondary IgA deficiency enhances susceptibility to Pseudomonas aeruginosa pneumonia. J. Clin. Investig..

[27] Brown R.L., Sequeira R.P., Clarke T.B. (2017). The microbiota protects against respiratory infection via GM-CSF signaling. Nat. Commun..

[28] Abt M.C., Osborne L.C., Monticelli L.A., Doering T.A., Alenghat T., Sonnenberg G.F., Paley M.A., Antenus M., Williams K.L., Erikson J. (2012). Commensal bacteria calibrate the activation threshold of innate antiviral immunity. Immunity.

[29] Ichinohe T., Pang I.K., Kumamoto Y., Peaper D.R., Ho J.H., Murray T.S., Iwasaki A. (2011). Microbiota regulates immune defense against respiratory tract influenza A virus infection. Proc. Natl. Acad. Sci. USA.

[30] Mirkov I., Demenesku J., Popov Aleksandrov A., Ninkov M., Glamoclija J., Kataranovski D., Kataranovski M. (2015). Strain differences in the immune mechanisms of resistance of immunocompetent rats to pulmonary aspergillosis. Immunobiology.

[31] Popovic D., Kulas J., Tucovic D., Popov Aleksandrov A., Glamoclija J., Sokovic Bajic S., Tolinacki M., Golic N., Mirkov I. (2023). Lung microbiota changes during pulmonary Aspergillus fumigatus infection in rats. Microbes Infect..

[32] Magoč T., Salzberg S.L. (2011). FLASH: Fast length adjustment of short reads to improve genome assemblies. Bioinformatics.

[33] Bokulich N.A., Subramanian S., Faith J.J., Gevers D., Gordon J.I., Knight R., Mills D.A., Caporaso J.G. (2013). Quality-filtering vastly improves diversity estimates from Illumina amplicon sequencing. Nat. Methods.

[34] Caporaso J.G., Kuczynski J., Stombaugh J., Bittinger K., Bushman F.D., Costello E.K., Fierer N., Peña A.G., Goodrich J.K., Gordon J.I. (2010). QIIME allows analysis of high-throughput community sequencing data. Nat. Methods.

[35] (2021). QIIME. http://qiime.org/scripts/assign_taxonomy.html.

[36] Quast C., Pruesse E., Yilmaz P., Gerken J., Schweer T., Yarza P., Peplies J., Glöckner F.O. SILVA Ribosomal RNA Gene Database. Max Planck Institute for Marine Microbiology, Bremen, Germany. https://www.arb-silva.de/.

[37] Segata N., Izard J., Waldron L., Gevers D., Miropolsky L., Garrett W.S., Huttenhower C. (2011). Metagenomic biomarker discovery and explanation. Genome Biol..

[38] Anderson M.E., Greenwald R.A. (1986). Tissue glutathione. The DTNB-GSSG reductase recycling assay for total glutathione (GSHþ1/2GSSG). Handbook of Methods for Oxygen Radical Research.

[39] Habig W.H., Pabst M.J., Jakoby W.B. (1974). Glutathione-S-Transferases: The first enzymatic step in mercapturic acid formation. J. Biol. Chem..

[40] Beutler E., Beutler E. (1982). Catalase. Red Cell Metabolism, a Manual of Biochemical Methods.

[41] Villacara A., Kumami K., Yamamoto T., Mrsulja B.B., Spatz M. (1989). Ischemic modification of cerebrocortical membranes: 5-hydroxytryptamine receptors, fluidity, and inducible in vitro lipid peroxidation. J. Neurochem..

[42] Lowry O.H., Rosebrough N.J., Farr A.L., Randall R.J. (1951). Protein measurement with the Folin phenol reagent. J. Biol. Chem..

[43] Bozeman P.M., Learn D.B., Thomas E.L. (1990). Assay of the human leukocyte enzymes myeloperoxidase and eosinophil peroxidase. J. Immunol. Methods.

[44] Raymond F., Ouameur A.A., Déraspe M., Iqbal N., Gingras H., Dridi B., Leprohon P., Plante P.L., Giroux R., Bérubé È. (2016). The initial state of the human gut microbiome determines its reshaping by antibiotics. ISME J..

[45] Rashidi A., Ebadi M., Rehman T.U., Elhusseini H., Nalluri H., Kaiser T., Holtan S.G., Khoruts A., Weisdorf D.J., Staley C. (2021). Gut microbiota response to antibiotics is personalized and depends on baseline microbiota. Microbiome.

[46] Cheng M., Ning K. (2019). Stereotypes About Enterotype: The Old and New Ideas. Genom. Proteom. Bioinform..

[47] Rao S., Kupfer Y., Pagala M., Chapnick E., Tessler S. (2011). Systemic absorption of oral vancomycin in patients with Clostridium difficile infection. Scand. J. Infect. Dis..

[48] Veirup N., Kyriakopoulos C. (2023). Neomycin. StatPearls.

[49] Moghaddam A., Graeser V., Westhauser F., Dapunt U., Kamradt T., Woerner S.M., Schmidmaier G. (2016). Patients’ safety: Is there a systemic release of gentamicin by gentamicin-coated tibia nails in clinical use?. Ther. Clin. Risk Manag..

[50] Watanakunakorn C. (1981). The antibacterial action of vancomycin. Rev. Infect. Dis..

[51] Lavelle A., Hoffmann T.W., Pham H.P., Langella P., Guédon E., Sokol H. (2019). Baseline microbiota composition modulates antibiotic-mediated effects on the gut microbiota and host. Microbiome.

[52] Hill D.A., Hoffmann C., Abt M.C., Du Y., Kobuley D., Kirn T.J., Bushman F.D., Artis D. (2010). Metagenomic analyses reveal antibiotic-induced temporal and spatial changes in intestinal microbiota with associated alterations in immune cell homeostasis. Mucosal Immunol..

[53] Yuan X., Zhou F., Wang H., Xu X., Xu S., Zhang C., Zhang Y., Lu M., Zhang Y., Zhou M. (2023). Systemic antibiotics increase microbiota pathogenicity and oral bone loss. Int. J. Oral. Sci..

[54] Shin N.R., Whon T.W., Bae J.W. (2015). Proteobacteria: Microbial signature of dysbiosis in gut microbiota. Trends Biotechnol..

[55] Fei N., Zhao L. (2013). An opportunistic pathogen isolated from the gut of an obese human causes obesity in germfree mice. ISME J..

[56] Morgan X.C., Tickle T.L., Sokol H., Gevers D., Devaney K.L., Ward D.V., Reyes J.A., Shah S.A., LeLeiko N., Snapper S.B. (2012). Dysfunction of the intestinal microbiome in inflammatory bowel disease and treatment. Genome Biol..

[57] Pärnänen K., Karkman A., Hultman J., Lyra C., Bengtsson-Palme J., Larsson D.G.J., Rautava S., Isolauri E., Salminen S., Kumar H. (2018). Maternal gut and breast milk microbiota affect infant gut antibiotic resistome and mobile genetic elements. Nat. Commun..

[58] Lin T.L., Shu C.C., Chen Y.M., Lu J.J., Wu T.S., Lai W.F., Tzeng C.M., Lai H.C., Lu C.C. (2020). Like cures like: Pharmacological activity of anti-inflammatory lipopolysaccharides from gut microbiome. Front. Pharmacol..

[59] García A., Salgado F., Solar H., González C.L., Zemelman R., Oñtate A. (1999). Some immunological properties of lipopolysaccharide from Acinetobacter baumannii. J. Med. Microbiol..

[60] Liu J., Hong W., Sun Z., Zhang S., Xue C., Dong N. (2026). The gut-lung axis: Effects and mechanisms of gut microbiota on pulmonary diseases. Front. Immunol..

[61] Maukonen J., Kolho K.L., Paasela M., Honkanen J., Klemetti P., Vaarala O., Saarela M. (2015). Altered fecal microbiota in paediatric inflammatory bowel disease. J. Crohns Colitis..

[62] Skalny A.V., Korobeinikova T.V., Morozova G., Menshikova I.V., Gritsenko V.A., Zhang F., Mak D.V., Guo X., Sotnikova T.I., Aschner M. (2025). Serum trace element and mineral levels and fecal microbiota in relation to cartilage damage in rheumatoid arthritis patients. J. Trace Elem. Med. Biol..

[63] Huang N., Hua D., Zhan G., Li S., Zhu B., Jiang R., Yang L., Bi J., Xu H., Hashimoto K. (2019). Role of Actinobacteria and Coriobacteriia in the antidepressant effects of ketamine in an inflammation model of depression. Pharmacol. Biochem. Behav..

[64] Alam M.T., Amos G.C.A., Murphy A.R.J., Murch S., Wellington E.M.H., Arasaradnam R.P. (2020). Microbial imbalance in inflammatory bowel disease patients at different taxonomic levels. Gut Pathog..

[65] Clavel T., Lepage P., Charrier C., Rosenberg E., DeLong E.F., Lory S., Stackebrandt E., Thompson F. (2014). The family *Coriobacteriaceae*. The Prokaryotes.

[66] Chiang-Ni C., Huang J.Y., Hsu C.Y., Lo Y.C., Chen Y.M., Lai C.H., Chiu C.H. (2024). Genetic diversity, biofilm formation, and Vancomycin resistance of clinical Clostridium innocuum isolates. BMC Microbiol..

[67] Dinakaran V., Mandape S.N., Shuba K., Pratap S., Sakhare S.S., Tabatabai M.A., Smoot D.T., Farmer-Dixon C.M., Kesavalu L.N., Adunyah S.E. (2019). Identification of specific oral and gut pathogens in full thickness colon of colitis patients: Implications for colon motility. Front. Microbiol..

[68] Nishino K., Nishida A., Inoue R., Kawada Y., Ohno M., Sakai S., Inatomi O., Bamba S., Sugimoto M., Kawahara M. (2018). Analysis of endoscopic brush samples identified mucosa-associated dysbiosis in inflammatory bowel disease. J. Gastroenterol..

[69] Said M.S., Tirthani E., Lesho E. (2024). Enterococcus Infections. StatPearls.

[70] Zhang W., Xu X., Cai L., Cai X. (2023). Dysbiosis of the gut microbiome in elderly patients with hepatocellular carcinoma. Sci. Rep..

[71] Li Z., Liu J., Li J., Zhou Z., Huang X., Gopinath D., Luo P., Wang Q., Shan D. (2025). Fusobacterium in the microbiome: From health to disease across the oral-gut axis and beyond. npj Biofilms Microbiomes.

[72] Sun L., Zhang X., Zhang Y., Zheng K., Xiang Q., Chen N., Chen Z., Zhang N., Zhu J., He Q. (2019). Antibiotic-induced disruption of gut microbiota alters local metabolomes and immune responses. Front. Cell Infect. Microbiol..

[73] Lange K., Buerger M., Stallmach A., Bruns T. (2016). Effects of antibiotics on gut microbiota. Dig. Dis..

[74] Singh N., Vats A., Sharma A., Arora A., Kumar A. (2017). The development of lower respiratory tract microbiome in mice. Microbiome.

[75] Huttenhower C., Kostic A.D., Xavier R.J. (2014). Inflammatory bowel disease as a model for translating the microbiome. Immunity.

[76] Kesavelu D., Jog P. (2023). Current understanding of antibiotic-associated dysbiosis and approaches for its management. Ther. Adv. Infect. Dis..

[77] Eladham M.W., Selvakumar B., Saheb Sharif-Askari N., Saheb Sharif-Askari F., Ibrahim S.M., Halwani R. (2024). Unraveling the gut-Lung axis: Exploring complex mechanisms in disease interplay. Heliyon.

[78] Al-Sadi R.M., Ma T.Y. (2007). IL-1beta causes an increase in intestinal epithelial tight junction permeability. J. Immunol..

[79] Yang R., Han X., Uchiyama T., Watkins S.K., Yaguchi A., Delude R.L., Fink M.P. (2003). IL-6 is essential for development of gut barrier dysfunction after hemorrhagic shock and resuscitation in mice. Am. J. Physiol. Gastrointest. Liver Physiol..

[80] Dessein R., Bauduin M., Grandjean T., Le Guern R., Figeac M., Beury D., Faure K., Faveeuw C., Guery B., Gosset P. (2020). Antibiotic-related gut dysbiosis induces lung immunodepression and worsens lung infection in mice. Crit. Care.

[81] Sacha P.T., Zaremba M.L., Jakoniuk P. (1999). The influence of antibiotics on phagocytic and bacteriocidal activity of rabbit peritoneal macrophages stimulated by filtrates of cultured t-lymphocytes. Med. Dosw. Mikrobiol..

[82] van de Veerdonk F.L., Netea M.G. (2010). T-cell Subsets and Antifungal Host Defenses. Curr. Fungal Infect. Rep..

[83] Cunha C., Gonçalves S.M., Duarte-Oliveira C., Leite L., Lagrou K., Marques A., Lupiañez C.B., Mesquita I., Gaifem J., Barbosa A.M. (2017). IL-10 overexpression predisposes to invasive aspergillosis by suppressing antifungal immunity. J. Allergy Clin. Immunol..

[84] Miyamoto M., Prause O., Sjöstrand M., Laan M., Lötvall J., Lindén A. (2003). Endogenous IL-17 as a mediator of neutrophil recruitment caused by endotoxin exposure in mouse airways. J. Immunol..

[85] Blagojević V., Kovačević-Jovanović V., Ćuruvija I., Petrović R., Vujnović I., Vujić V., Stanojević S. (2018). Rat strain differences in peritoneal immune cell response to selected gut microbiota: A crossroad between tolerance and autoimmunity?. Life Sci..

[86] Arumugam M., Raes J., Pelletier E., Le Paslier D., Yamada T., Mende D.R., Fernandes G.R., Tap J., Bruls T., Batto J.M. (2011). Enterotypes of the human gut microbiome. Nature.

[87] Choi S.I., Kim N., Nam R.H., Park J.H., Nho H., Yu J.E., Song C.H., Lee S.M., Lee D.H. (2021). Fecal microbial enterotypes differentially respond to a high-fat diet based on sex in Fischer-344 rats. J. Cancer Prev..

[88] Kolzhetsov N., Markelova N., Frolova M., Alikina O., Glazunova O., Safonova L., Kalashnikova I., Yudin V., Makarov V., Keskinov A. (2024). Enterotype-Dependent Probiotic-Mediated Changes in the Male Rat Intestinal Microbiome In Vivo and In Vitro. Int. J. Mol. Sci..

[89] Knights D., Ward T.L., McKinlay C.E., Miller H., Gonzalez A., McDonald D., Knight R. (2014). Rethinking “enterotypes”. Cell Host Microbe.

[90] Jeffery I.B., Claesson M.J., O’Toole P.W., Shanahan F. (2012). Categorization of the gut microbiota: Enterotypes or gradients?. Nat. Rev. Microbiol..

[91] Bah Y.R., Baba K., Mustafa D.N.A.B., Watanabe S., Takeda A.K., Yamashita T., Kasahara K. (2025). Bacteroides- and Prevotella-enriched gut microbial clusters associate with metabolic risks. Gut Pathog..

[92] Wexler H. (2007). Bacteroides: The good, the bad, and the nitty-gritty. Clin. Microbiol. Rev..

[93] Jean S., Wallace M.J., Dantas G., Burnham C.D. (2022). Time for some group therapy: Update on identification, antimicrobial resistance, taxonomy, and clinical significance of the Bacteroides fragilis group. J. Clin. Microbiol..

[94] Shin J.H., Tillotson G., MacKenzie T.N., Warren C.A., Wexler H.M., Goldstein E.J.C. (2024). Bacteroides and related species: The keystone taxa of the human gut microbiota. Anaerobe.

[95] Fansler R.T., Wu Y., Zhu W. (2026). Friend or foe? Contextualizing Bacteroides through the lens of niche remodeling. Trends Microbiol..

[96] Sayols-Baixeras S., Dekkers K.F., Baldanzi G., Jönsson D., Hammar U., Lin Y.T., Ahmad S., Nguyen D., Varotsis G., Pita S. (2023). *Streptococcus* Species Abundance in the Gut Is Linked to Subclinical Coronary Atherosclerosis in 8973 Participants from the SCAPIS Cohort. Circulation.

[97] Li S., Guo J., Liu R., Zhang F., Wen S., Liu Y., Ren W., Zhang X., Shang Y., Gao M. (2022). Predominance of Escherichia-Shigella in gut microbiome and its potential correlation with elevated level of plasma tumor necrosis factor alpha in patients with tuberculous meningitis. Microbiol. Spectr..

[98] Reygaert W.C. (2018). An overview of the antimicrobial resistance mechanisms of bacteria. AIMS Microbiol..

[99] Wu G.D., Chen J., Hoffmann C., Bittinger K., Chen Y.Y., Keilbaugh S.A., Bewtra M., Knights D., Walters W.A., Knight R. (2011). Linking long-term dietary patterns with gut microbial enterotypes. Science.

[100] Lim M.Y., Rho M., Song Y.M., Lee K., Sung J., Ko G. (2014). Stability of gut enterotypes in Korean monozygotic twins and their association with biomarkers and diet. Sci. Rep..

[101] Ogata T., Arima H., Kawazoe M., Baba Y. (2025). Association between enterotypes of the gut microbiota and features of ischemic stroke. Sci. Rep..

[102] Le Chatelier E., Nielsen T., Qin J., Prifti E., Hildebrand F., Falony G., Almeida M., Arumugam M., Batto J.M., Kennedy S. (2013). Richness of human gut microbiome correlates with metabolic markers. Nature.

[103] Hildebrand F., Nguyen T.L., Brinkman B., Yunta R.G., Cauwe B., Vandenabeele P., Liston A., Raes J. (2013). Inflammation-associated enterotypes, host genotype, cage and inter-individual effects drive gut microbiota variation in common laboratory mice. Genome Biol..

